# Declining Responsiveness of *Plasmodium falciparum* Infections to Artemisinin-Based Combination Treatments on the Kenyan Coast

**DOI:** 10.1371/journal.pone.0026005

**Published:** 2011-11-10

**Authors:** Steffen Borrmann, Philip Sasi, Leah Mwai, Mahfudh Bashraheil, Ahmed Abdallah, Steven Muriithi, Henrike Frühauf, Barbara Schaub, Johannes Pfeil, Judy Peshu, Warunee Hanpithakpong, Anja Rippert, Elizabeth Juma, Benjamin Tsofa, Moses Mosobo, Brett Lowe, Faith Osier, Greg Fegan, Niklas Lindegårdh, Alexis Nzila, Norbert Peshu, Margaret Mackinnon, Kevin Marsh

**Affiliations:** 1 Kenya Medical Research Institute/Wellcome Trust Research Programme, Kilifi, Kenya; 2 Heidelberg University School of Medicine, Dept. of Infectious Diseases, Heidelberg, Germany; 3 Department of Clinical Pharmacology, Muhimbili University of Health and Allied Sciences, Dar es Salaam, Tanzania; 4 Mahidol-Oxford Tropical Medicine Research Unit, Faculty of Tropical Medicine, Mahidol University, Bangkok, Thailand; 5 Division of Malaria Control, Ministry of Health, Nairobi, Kenya; 6 District Office, Ministry of Health, Kilifi, Kenya; 7 Centre for Tropical Medicine, Nuffield Department of Clinical Medicine, University of Oxford, CCVTM, Oxford, United Kingdom; Walter & Eliza Hall Institute, Australia

## Abstract

**Background:**

The emergence of artemisinin-resistant *P. falciparum* malaria in South-East Asia highlights the need for continued global surveillance of the efficacy of artemisinin-based combination therapies.

**Methods:**

On the Kenyan coast we studied the treatment responses in 474 children 6–59 months old with uncomplicated *P. falciparum* malaria in a randomized controlled trial of dihydroartemisinin-piperaquine *vs.* artemether-lumefantrine from 2005 to 2008. (ISRCTN88705995)

**Results:**

The proportion of patients with residual parasitemia on day 1 rose from 55% in 2005–2006 to 87% in 2007–2008 (odds ratio, 5.4, 95%CI, 2.7–11.1; *P*<0.001) and from 81% to 95% (OR, 4.1, 95%CI, 1.7–9.9; *P* = 0.002) in the DHA-PPQ and AM-LM groups, respectively. In parallel, Kaplan-Meier estimated risks of apparent recrudescent infection by day 84 increased from 7% to 14% (*P* = 0.1) and from 6% to 15% (*P* = 0.05) with DHA-PPQ and AM-LM, respectively. Coinciding with decreasing transmission in the study area, clinical tolerance to parasitemia (defined as absence of fever) declined between 2005–2006 and 2007–2008 (OR body temperature >37.5°C, 2.8, 1.9–4.1; *P*<0.001). Neither *in vitro* sensitivity of parasites to DHA nor levels of antibodies against parasite extract accounted for parasite clearance rates or changes thereof.

**Conclusions:**

The significant, albeit small, decline through time of parasitological response rates to treatment with ACTs may be due to the emergence of parasites with reduced drug sensitivity, to the coincident reduction in population-level clinical immunity, or both. Maintaining the efficacy of artemisinin-based therapy in Africa would benefit from a better understanding of the mechanisms underlying reduced parasite clearance rates.

**Trial Registration:**

Controlled-Trials.com ISRCTN88705995

## Introduction

Over the last few decades the global spread of parasite resistance to key antimalarial drugs such as chloroquine and pyrimethamine has been a challenge for malaria control programs based primarily on prompt and effective treatment [1]–[3]. The introduction of highly efficacious artemisinin-based combination treatments (ACT) as first-line treatment in most malaria endemic countries has contributed to recent notable reversals of trends in childhood morbidity and mortality [4], [5]. Because of the prominent value of ACTs in current malaria control programs, the emergence of parasite resistance to artemisinins and the associated compromised efficacy of ACTs would pose a major public health problem. The recently reported emergence of artemisinin-resistant malaria characterized by slow initial parasite clearance and high rates of recrudescent infections in Western Cambodia and, possibly, other countries South East Asia is therefore of great concern [6]–[9].

Using data from a randomized controlled clinical trial, we performed a post-hoc analysis of the *in vivo* response to two ACT regimens, namely dihydroartemisinin-piperaquine (DHA-PPQ) and artemether-lumefantrine (AM-LM) over time. The study was conducted from 2005 to 2008, coinciding with the introduction of artemether-lumefantrine (Coartem™) as the exclusive first-line treatment for all presumptive cases of uncomplicated *P. falciparum* malaria in Kilifi District, Coast Province, Kenya in 2006.

## Methods

### Study site

The study was conducted at the Pingilikani study site [10], [11]. Malaria transmission in the area is perennial but with peaks trailing typically two annual rainy seasons [12]. The parasite positivity rate in outpatients has declined precipitously from 2003 to 2005 (own unpublished data and [12]). The study was approved by the National KEMRI Ethical Review Committee, Kenya; the Oxford Tropical Research Ethics Committee, UK; and the Ethics Committee, Heidelberg University School of Medicine, Germany. The protocol for this trial and supporting CONSORT checklist are available as supporting information; see Checklist S1 and Protocol S1.

### Study design and sample size

This is a detailed analysis of treatment response rates according to year of enrollment in a non-inferiority randomized controlled trial that evaluated the efficacy of DHA-PPQ vs. AM-LM in the treatment of children with uncomplicated *P. falciparum* malaria in Kilifi, Kenya (Controlled Trials Registry number, ISRCTN88705995). The primary efficacy endpoint was the 28-day cure rate adjusted for reinfection (defined as clearance of asexual parasites by day 7 and absence of PCR-confirmed recrudescence of primary infection). Assuming a cure rate of 95% with AM-LM and a 5% drop-out rate, we calculated that 250 patients per arm would provide 80% power to test a 5% non-inferiority margin with a 97.5% one-sided confidence interval.

### Enrollment of patients

We enrolled pediatric outpatients aged 6–59 months with uncomplicated *P. falciparum* malaria who met the following selection criteria: reported or documented fever ≥37.5°C, *P. falciparum* mono-infection, microscopically determined peripheral asexual parasite density of 2,000–200,000/µL, body weight >5 kg and signed informed consent by parent or legal guardian. We excluded patients with known allergies, severe malaria or danger signs [13], participation in an investigational drug study within previous 30 days, ECG abnormalities requiring urgent management, other relevant clinical conditions or severe acute malnutrition. A randomization list was generated by an independent off site contract research organization (CRO). Sealed envelopes containing treatment allocation were used to randomize eligible patients to treatment with DHA-PPQ or AM-LM. The randomization ratio was 2∶1 (DHA-PPQ∶AM-LM) for patients enrolled in 2005–2006 and reversed to 1∶2 in 2007–2008 to accommodate a multicenter trial analysis [14] that required 2∶1 randomization for patients enrolled in 2005–2006 while achieving an overall balanced 1∶1 randomization.

### Study drug and administration

Study drugs were administered orally with food or drinks under direct supervision. For children <2 years old tablets were crushed, mixed with 50 ml water, and administered as slurry. DHA-PPQ was given 24-hourly for 3 days at single target doses of 2.25 mg of DHA/kg body weight and 18 mg of PPQ/kg (formulated as pediatric or adult strength fixed dose combinations of 20/160 mg and 40/320 mg, respectively; Eurartesim™, SigmaTau, Italy). Doses were rounded up to the nearest half tablet. AM-LM was administered as whole tablets according to the manufacturer's instructions in 6 doses over 3 days (0, 8, 24, 36, 48, and 60 hours) at mean target doses of 2 mg of AM/kg and 12 mg of LM/kg (Coartem™, Novartis, Switzerland). Participants who vomited or rejected the study drug within 30 min received a second full dose, and those who vomited or rejected the study drug after 30 min but within 1 h received a second half dose. Vomiting or rejecting the second dose led to withdrawal from the study and administration of rescue medication. Stability tests that were performed by the drug manufacturers upon request confirmed that titer and degradation products of study drugs were still within stringent regulatory specifications in 2009, >10 months after completion of the study.

### Study flow and clinical procedures

During the 3-day treatment phase, patients were admitted to the KEMRI research ward in Pingilikani to ensure strict adhesion to dosing intervals. Patients were seen by study clinicians on days 0, 1, 2 and 3 and then for weekly follow-up visits until day 63 and finally on day 84 for collecting medical history, vital signs, malaria blood smears, and adverse events. Giemsa-stained malaria slides were read blinded by the same microscopist throughout the study according to KEMRI standard operating procedures.

### Genotyping

To distinguish recrudescent from new infections and to determine the multiplicity of infection (MOI) index, matched pairs of parasite isolates obtained at baseline and recurrence were compared using RFLP-based genotyping analysis of repeat length polymorphisms in the *MSP2* gene (PFB0300c) [15]. Results were verified by genotyping additional loci (*MSP1* and *GLURP*) or semi-automated capillary electrophoresis-based analysis of fluorescence-labeled *MSP2* PCR products [16]. Recrudescence was defined as persistence of at least one baseline clone. Genomic copy number variants at the *MDR1* locus (PFE1150w) were determined by quantitative real-time PCR using the β-tubulin gene (PF10_0084) as internal control and 3D7 (1 copy) and Dd2 (2 copies) reference strains for calibration [17].

### Pharmacokinetic measurements

Serum samples collected on day 7 were analyzed for LM by solid-phase extraction and liquid chromatography (LC) with UV detection as described previously [18], PPQ and its stable isotope labeled internal standard were analyzed using high throughput LC-MS/MS on an ABI 5000 triple quadrupole mass spectrometer (Applied Biosystems/MDS SCIEX, Foster City, USA), with a TurboV ionization source interface operated in the positive ion mode [19]. The lower limit of quantification (LLOQ) for PPQ and LM were set to 1.5 ng/ml and 50 ng/ml, respectively.

### Parasite adaptation and chemosensitivity testing

Parasite isolates were adapted to *in vitro* culture according to standard protocols [10]. We determined the concentrations of DHA, PPQ and LM required to inhibit the *in vitro* growth of parasite isolates by 50% (IC_50_) compared to unexposed controls using regression analysis of the dose-response curves from duplicate ^3^H-hypoxanthine uptake 72-hour exposure experiments [10]. Two reference strains (V1S, a multidrug-resistant strain, and 3D7, a drug-sensitive strain) were used as controls.

### Anti-parasitic antibody responses

We used an established ELISA protocol to measure concentrations of antibodies against parasite schizont extract (A4 strain) [20]. Hyper-immune sera from Kenyan donors and sera from unexposed European individuals were run in duplicate on each plate as positive and negative controls, respectively.

### Statistical analysis

**Figure 1:**
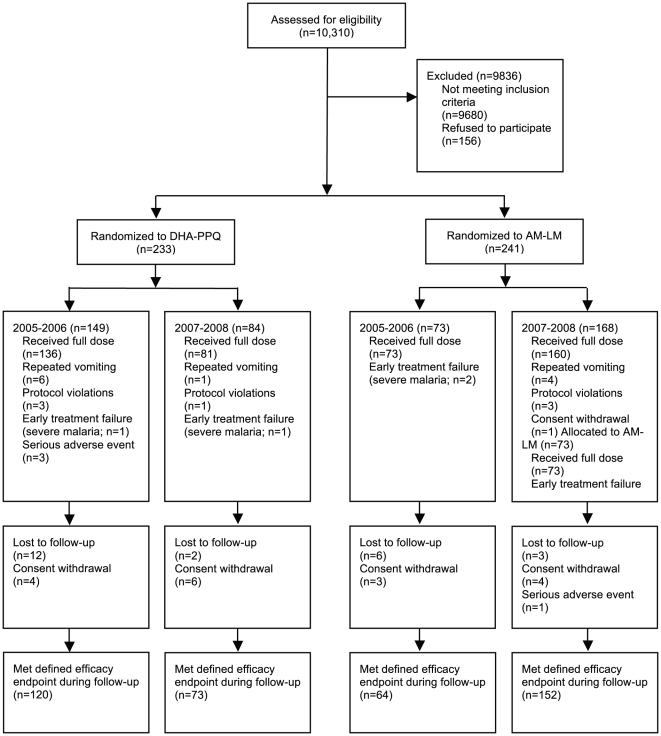
Study profile.

As a measure of drug efficacy, we computed the day 1 parasite reduction ratio (PRR_D1_) as the log_10_ quotient of baseline and day 1 parasitemias (after setting parasitemias below the microscopic detection threshold on day 1 to 10/µL). We also analyzed the probability that parasites were detected by microscopy or not on day 1 by binomial logistic regression for the influences of period of enrolment (2005–2006 vs. 2007–2008), log_10_ baseline parasitemia, treatment (AM-LM vs. DHA-PPQ), dose per body weight, patient age, number of previous malaria episodes and anti-schizont antibody levels fitting each of these separately in univariable analyses and then combined in one multivariable analysis if they remained significant at the *P*≤0.2 level. Similarly, we analyzed percentage reductions of parasite densities from baseline calculated by dividing baseline densities with densities at day 1 or 2 multiplied by 100. Parasite and fever clearance times were estimated by parametric survival analysis of the time from baseline to the first of two consecutive negative blood smears or temperature measurements <37.5°C, respectively. Risk of recrudescent primary or secondary (re-) infections was assessed by the Kaplan-Meier (KM) method for survival data. For these analyses, patients who did not meet a study endpoint (either absence or recurrence of parasitemia from day 7 to day 84) were censored at the last visit before dropout (Fig. 1). Cox proportional hazards model was used to estimate hazards ratios (HR). Data on previous malaria episodes were obtained by matching subjects to a passive outpatient surveillance system operated at Pingilikani since 2003. Other traits were analyzed by either *t*-test or linear regression assuming normal distributions, by Kruskal-Wallis equality-of-populations rank test assuming non-parametric distribution or, if proportion or count data, by Fisher's exact test and Poisson logistic regression, respectively. All analyses were performed with Stata 11.0 (StataCorp, College Station, TX).

## Results

### Study cohort

Between September 2005 and April 2008 we enrolled 474 patients. Enrollment was temporarily suspended between July 2006 and March 2007. 450 patients received a full treatment course (Fig. 1). Repeated vomiting occurred in 7/233 (3%) and 4/241 (2%) patients in the DHA-PPQ and AM-LM groups, respectively (*P*>0.3) (Fig. 1). Early treatment failure due to severe malaria occurred in 3 patients receiving DHA-PPQ and 1 patient receiving AM-LM group (Fig. 1). Table 1 details baseline characteristics of patients by treatment group and period of enrollment. In a pooled analysis of both treatment groups, patients enrolled in 2007–2008 were between 5 to 7 months older (*P*<0.05), weighed about 1 kg more (*P*<0.05), had higher parasitemias (*P* = 0.03) and axillary temperatures (*P*<0.001), increased number of parasite clones as determined by *MSP2* allele typing (*P*<0.01) and higher hemoglobin concentration (*P* = 0.03 in AM-LM group only). Other characteristics, notably platelet concentrations [21], were similar between enrollment periods.

**Table 1:** Baseline characteristics of patients.

Characteristics	Dihydroartemisinin-piperaquine					Artemether-lumefantrine				
	2005–2006(n = 149)		2007–2008(n = 84)		P value	2005–2006(n = 73)		2007–2008(n = 168)		P value
**Demographic**										
Female/male gender	80/69		45/39		1.0	33/40		70/96		0.7
Median age (range) in years	2.1	(0.5–4.9)	2.5	(0.6–4.9)	0.03	2.2	(0.5–4.8)	2.8	(0.5–4.9)	<0.001
Median weight (range) in kg	10.1	(6.3–19.4)	11.1	(6.2–20.7)	0.04	10.5	(6.4–17.0)	11.6	(6.4–18.0)	<0.001
**Clinical**										
Median axillary temperature (range) in °C	37.3	(36.0–40.1)	38.0	(36.2–40.5)	<0.001	37.3	(35.6–39.4)	37.9	(35.9–41.4)	<0.001
Mean heart rate (SD) in bpm	143	(25)	143	(20)	0.7	149	(21)	147	(23)	0.8
**Parasitological**										
Mean number of previous *P. falciparum* malaria episodes (N; range)	2.5	(69; 0–15)	1.5	(45; 0–7)	0.2	1.6	(29; 0–7)	2.0	(91; 0–12)	0.5
Median asexual *P. falciparum* density (range) in parasites/µL	34,600	(2,200–218,000)	36,600	(2,200–290,000)	0.8	44,500	(2,400–350,000)	57,600	(2,100–862,000)	0.07
Proportion of patients with gametocytes (%)	4/149	(3%)	1/84	(1%)	0.7	1/73	(1%)	2/167	(1%)	1.0
Multiplicity of infection (number of clones, SD)	2.1	(1.2)	2.8	(1.2)	0.003	1.7	(0.7)	2.7	(1.2)	0.004
Infections with >1 genomic copy of *MDR1*	0/45	(0%)	0/28	(0%)	1.0	0/23	(0%)	2/62	(3%)	1.0
**Hematology**										
Mean hemoglobin concentration (SD) in g/dL	9.0	(1.6)	9.2	(1.8)	0.4	8.8	(1.3)	9.2	(1.6)	0.03
Median neutrophil count (range) in 10^3^ cells/µL	3.5	(0.6–15.5)	3.3	(1.3–16.5)	0.9	3.6	(1.0–17.5)	3.7	(0.5–13.1)	0.6
Median platelet count (range) in 10^3^ cells/µL	166	(14–557)	154	(45–420)	0.7	114	(9–483)	142	(2–445)	0.8
**Clinical chemistry**										
Medium serum ALT level (range) in IU/L	22	(8–330)	21	(9–173)	0.7	23	(9–230)	23	(8–844)	0.5
Medium serum creatinine (range) in µmol/L	41	(3–73)	40	(24–67)	0.9	39	(22–58)	40	(20–71)	0.3
Medium serum bilirubin (range) in µmol/L	13	(2–142)	12	(2–58)	0.1	16	(2–71)	16	(1–126)	0.8

### Parasitological and clinical treatment responses

Treatment with DHA-PPQ cleared parasites faster than AM-LM (means of 41 hours *vs.* 48 hours, respectively; *P*<0.001) resulting also in prompter clearance of fever (means of 27 hours *vs.* 30 hours respectively; *P*<0.001). By day 28, Kaplan-Meier (KM)-estimated PCR-adjusted rates of recrudescent primary infections were 1% (95% CI, 0–4) and 1% (95% CI, 0–4) in the DHA-PPQ and AM-LM groups, respectively (HR = 0.9; 95% CI, 0.2–4.7). By day 84 we noted considerably higher PCR-adjusted recrudescence rates with both DHA-PPQ (10%; 95% CI, 6–15) and AM-LM (13%; 95% CI, 8–18) (HR, 1.3; 95% CI, 0.7–2.4). By day 84 reinfections occurred in 39% (KM 95% CI, 33–47) and 42% (KM 95% CI, 35–49) of children treated with DHA-PPQ and AM-LM groups, respectively (*P* = 0.7), with no difference in median time to reinfection (Table 2).

**Table 2:** Treatment responses in pediatric patients treated with artemisinin-based combination chemotherapies.

Treatment response parameters	Dihydroartemisinin-piperaquine					Artemether-lumefantrine				
	2005–2006(n = 149)		2007–2008(n = 84)		P value	2005–2006(n = 73)		2007–2008(n = 168)		P value
**Initial responses to treatment**										
Mean time to parasite clearance (95% CI) in hours	37.8	(35.6–40.0)	46.0	(43.8–48.1)	0.002	45.0	(42.3–47.8)	49.8	(48.2–51.3)	0.02
Parasite prevalence by day 1 (%)	78/142	(55%)	73/84	(87%)	<0.001	59/73	(81%)	155/164	(95%)	0.002
Parasite prevalence by day 2 (%)	6/142	(4%)	4/83	(5%)	1.0	5/73	(7%)	21/163	(13%)	0.2
Parasite prevalence by day 3 (%)	0/142	(0%)	0/82	(0%)	-	0/73	(0%)	0/163	(0%)	-
Median log_10_ day 1 parasite reduction ratio (95% CI)	2.5	(2.3–2.6)	1.8	(1.4–2.0)	<0.001	1.9	(1.6–2.2)	1.4	(1.3–1.6)	<0.001
- in children aged <2.5 yrs	2.4	(2.2–2.5)	1.7	(1.4–1.8)	<0.001	2.2	(2.0–2.4)	1.4	(1.3–1.6)	<0.001
- in children aged ≥2.5 yrs	2.3	(2.2–2.5)	1.4	(1.3–1.6)	<0.001	2.2	(2.1–2.3)	1.4	(1.3–1.7)	<0.001
% of baseline parasite density										
Median day 1 (range)	0.2%	(0%–27%)	1.6%	(0%–110%)	<0.001	1.3%	(0%–25%)	4.1%	(0%–1,100%)	<0.001
Median day 2 (range)	0%	(0%–1.3%)	0%	(0%–0.5%)	0.8	0%	(0%–0.4%)	0%	(0%–8%)	0.1
Mean time to fever clearance (95% CI) in hours	25.7	(24.7–26.7)	28.0	(26.0–30.1)	<0.001	27.3	(25.4–29.2)	30.7	(29.0–32.4)	0.002
**Risk of recurrent parasitemia**										
Day 28 KM estimate of recrudescent primary infections (95% CI) in %	1.1	(0.4–5.9)	1.3	(0.2–8.7)	1.0	0	-	1.1	(0.6–5.8)	0.5
Day 84 KM estimate of recrudescent primary infections (95% CI) in %	7.2	(3.6–14.1)	14.4	(7.7–26.2)	0.1	5.8	(1.9–17.3)	15.4	(10.3–22.7)	0.05
Day 84 KM estimate of reinfections (95% CI) in %	35.7	(27.9–44.8)	45.0	(34.4–57.1)	0.2	38.5	(27.8–51.6)	42.9	(35.3–51.4)	0.6
Median time to reinfection (range) in days	42	(21–84)	42	(21–84)	0.7	42	(21–84)	49	(19–84)	0.8
Median hemoglobin recovery^1^ (range) in g/dL	1.0	(−2.2–6.0)	0.9	(−2.3–5.8)	0.3	1.1	(−1–5.4)	1.0	(−2.1–5.4)	0.7
Gametocyte carrier rate (%)^2^	4/136	(3%)	1/76	(1%)	0.7	1/73	(1%)	1/154	(0.6%)	0.5

1 Change of hemoglobin blood concentration from baseline to day 28.

2 Cumulative rate from day 7 to day 84.

**Figure 2:**
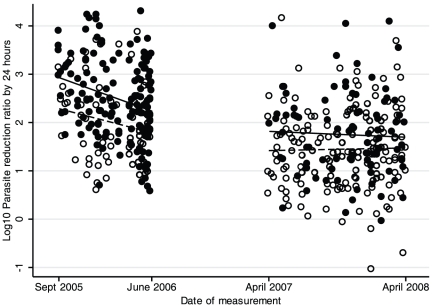
Scatter plot of day 1 parasite reduction ratios (PRR_D1_) in children with uncomplicated *P. falciparum* malaria by treatment group over time. Solid and hollow circles represent PRR_D1_s from patients treated with DHA-PPQ and AM-LM, respectively. Solid and dashed lines indicate linear regression lines for the two treatment groups, respectively. In 2007–2008 an expansion of parasitemia after start of treatment was observed in some patients treated with AM-LM.

When comparing treatment responses in patients enrolled in 2005–2006 *vs.* 2007–2008 in a post-hoc analysis we observed a striking increase in the proportion of children with detectable parasitemia one day after initiation of treatment (day 1 parasite prevalence rate, PPR_D1_; Table 2). This proportion rose from 55% to 87% (odds ratio, 5.4, 95% CI, 2.7–11.1; *P*<0.001) in the DHA-PPQ group and from 81% to 95% (OR, 4.1, 95% CI, 1.7–9.9; *P* = 0.002) in the AM-LM group. Median day 1 parasite reduction ratio in the DHA-PPQ group dropped by 78% (4.6-fold lower) and in the AM-LM group by 69% (3.2-fold lower) between the 2005–2006 and 2007–2008 enrollment periods (*P*<0.001 by logistic regression) and the same effect was observed within age groups (Fig. 2 and Table 2). These changes in initial parasite responses were accompanied by significantly prolonged mean times to fever clearance (Table 2; *P*<0.002) and a more than two fold risk of apparent recrudescent primary infections by day 84 (Table 2; *P* = 0.01 when pooling data across treatment groups). Reinfection rates by day 84, which could have confounded PCR-based classification of recrudescent primary infections [22], were high but did not change over time (*P*>0.2; Table 2). Gametocyte carrier rates during follow-up were low and independent of study period (Table 2). Of note, median times to reinfection, a sensitive measure for the post-treatment suppressive efficacy of the long-half life companion drugs PPQ and LM remained stable throughout the study (Table 2).

### Pharmacokinetic parameters of drug exposure

A total of 105 and 101 serum samples obtained on day 7 from patients who received either DHA-PPQ or AM-LM were analyzed for piperaquine and lumefantrine, respectively. Day 7 serum concentrations did not differ between the two study periods (all p values>0.3) (Table S1). Since samples were unavailable for directly determining plasma concentrations of artemisinin derivatives, we compared body weight-adjusted doses of DHA and AM as proxy of plasma drug exposure, respectively. Although median doses of both DHA and AM were slightly lower in 2007–2008 compared to 2005–2006 (Table S1), day 1 parasite reduction ratios dropped independently in children above or below average body weight-adjusted doses (Table S1).

### Clinical, epidemiological and serological parameters of immunity

**Figure 3:**
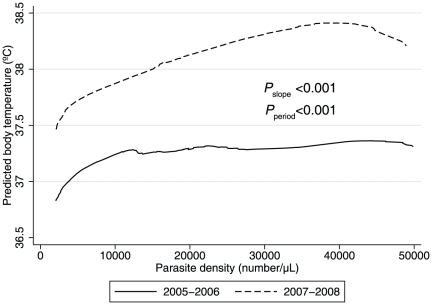
Locally weighted regression (LOWESS) lines of baseline body temperature by baseline asexual parasite density. The dashed line for patients enrolled in 2007–2008 indicates a substantial reduction in clinical tolerance of patients to high parasitemias as compared to patients enrolled in 2005–2006 (solid line).

We observed a reduced tolerance to any parasitemia but in particular, high-density parasitemia in children enrolled in the second compared with the first study period (Fig. 3; OR for body temperature >37.5°C, 2.8, 1.9–4.1; *P*<0.001). This apparent loss in population-level clinical immunity could not be explained by lower numbers of previous recorded exposure in study participants (2.2 *vs.* 1.9 previous malaria episodes in 2005–2006 and 2007–2008, respectively; *P* = 0.4) or serological indices of exposure (median OD of anti-schizont antibodies of 0.71 *vs.* 0.62 in 2005–2006 *vs.* 2007–2008, respectively; *P* = 0.3) (Table 1 and Fig. S1).

### Multivariable analysis of parasite elimination rate

In a multivariable logistic regression model only enrollment in 2007–2008, baseline parasitemia, body temperature and treatment group independently predicted PPR_D1_ (OR>1.9 and *P*<0.001) (Table 3). Anti-schizont antibody levels did not predict PPR_D1_ in a univariable analysis (Table 3).

**Table 3:** Univariable and multivariable analysis of the risk of residual parasitemia on day 1 after initiation of treatment.

Variable (Unit)	Univariable analysis				Multivariable model		
					Wald χ^2^ = 87.7		
	Odds ratio	95% CI	Wald χ^2^	P value	Odds ratio	95% CI	P value
Period (2007–2008)	6.5	(3.8–11)	46.9	<0.001	4.4	(2.2–8.7)	<0.001
Baseline parasitemia (log_10_ of parasites/µL)	6.3	(3.9–10)	59.1	<0.001	6.7	(3.6–12)	<0.001
Tympanic temperature (°C)	2.5	(1.9–3.3)	38.9	<0.001	1.9	(1.4–2.5)	<0.001
Treatment (AM-LM)	4.6	(2.8–7.7)	34.3	<0.001	3.4	(1.8–6.6)	<0.001
DHA or artemether daily dose (mg/kg body weight)	1.5	(1.2–2.0)	9.0	0.003	0.8	(0.4–1.5)	0.5
Patient age (1 year)	1.2	(0.9–1.4)	2.1	0.2	1.0	(0.8–1.3)	0.8
Previous malaria episodes (Number)	1.1	(0.9–1.2)	0.7	0.4	-	-	-
Anti-schizont antibody levels (OD)	0.8	(0.3–1.8)	0.4	0.5	-	-	-

To further dissect potential associations of covariates with the decline through time of parasite elimination rate, risk of parasitemia on day 1 (PPR_D1_) was analyzed for time differences with baseline parasitemia as a continuous covariate. When this was done, the differences between time periods remained, particularly for those patients with low baseline parasitemia (Fig. S2A) (interaction term *P* = 0.06). Thus the least parasitized children experienced the greatest decrease in efficacy through time. The risk of parasitemia on day 1 was also significantly higher in children enrolled in 2007–2008 after adjusting for patient age (Fig. S2B) and body weight-adjusted DHA dose (Fig. S2C) (*P*_period_<0.005 for all comparisons). Similar results were obtained for AM-LM (Fig. S2D–F).

### *In vitro* drug responses and genetic markers

Baseline IC_50_ responses for DHA (n = 59), PPQ (n = 53) and LM (n = 60) were not associated with PPR_D1_ (data not shown; *P*>0.2 by logistic regression). Paradoxically, median DHA IC_50_ values dropped from 2.2 nM in 2005–2006 (n = 39) to 0.8 nM in 2007–2008 (n = 20) (*P*<0.001; Fig. S3A). *In vitro* activities of PPQ or LM did not change over the same period of time (Fig. S3B, C; *P*>0.3).

Only 2/84 patients at baseline and 1/52 patients at recurrence (all in second period) were found to harbor infections with >1 copy of *MDR1* per parasite genome (Table 1).

## Discussion

Here we report a significant decline of early response rates of *P. falciparum* infections to treatment with ACTs after three years of their use in a randomized controlled clinical trial and less than two years after the introduction of AM-LM as first-line treatment in the Coast Province of Kenya. The parasite prevalence rate on day 1, which is primarily determined by the fast acting artemisinin component of ACTs [23], increased from 55% to 87% in patients receiving DHA-PPQ. In parallel, risk of recrudescences until day 84 doubled, although potential misclassification of re- infections detected after day 42 as recrudescent infections due to genotyping artifacts [22], [24] calls for a careful interpretation of these data. Similar results were obtained in patients treated with AM-LM. This is the first report of a decline in the responsiveness to ACTs from a malaria-endemic area outside of Southeast Asia. It is important to note that the magnitude of the decreased response rates observed here is much smaller than the substantially delayed clearance of *P. falciparum* infection reported from Western Cambodia (times to parasite clearance of 48 hours *vs.* 84 hours, respectively) [6], and does not meet current WHO working criteria for emerging *in vivo* artemisinin resistance [25]. In particular, all infections in our study treated with full and correctly dosed ACT regimens cleared before day 3. However, the observed increase in the presence of parasites after one day of treatment is reminiscent of incremental changes reported from the Thai-Burmese border in 2001 after 6 years of regulated use of artemisinin combinations in that region [26].

What factors could plausibly have caused the observed drop in early treatment responses measured at peak drug exposure? On average children enrolled in 2007–2008 were 6 months older (*P*<0.05), had higher body temperatures (0.6°C; *P*<0.001), and had 1.3-fold higher baseline parasite densities (*P*<0.05). These differences are most likely to be a consequence of decreased transmission in the study area that occurred mainly before 2005 [12], [27]. The latter two parameters, which indicate substantially less clinical immunity in children who grew up after the sharp drop in malaria transmission, were independent strong predictors of the risk of residual parasitemia on day 1 (PPR_D1_) (Table 3 and Fig. S2A, D) thus raising the possibility that the decline through time in parasite clearance rates was directly due to a loss of immunity rather than to a change in the level of sensitivity to drugs in the parasite population. However, levels of antibodies against crude blood stage extract did not predict parasite clearance rates and remained stable through time, possibly indicating that these antibodies are insensitive for detecting significant changes in parasite growth-limiting immunity. Moreover, parasite density at the time of enrollment, which is likely to be a good indicator of immunity, did not explain the differences between the time periods in PPR_D1_. Both patient age as a demographic correlate of progressively acquired immunity and specific antibody responses have shown consistent associations with risk of recrudescent primary infections [22], [28]–[32]. The contribution, if any, of acquired antiparasitic immune responses to parasite clearance during peak drug exposure, as observed in this study, is less evident, however [6], [28]: associations between patient age and early response parameters have been reported for weak or failing drugs [28], but not, including in this study, for highly active artemisinin drugs [6]. More studies of the relative contributions of immunity, parasite resistance and their interactions to the immediate efficacy of drugs are required.

Is it possible to quantify the extent, if any, to which changes in parasite sensitivity to the drugs could have contributed to the observed progressive reduction in responsiveness to treatment (Fig. 2)? We failed to find *in vitro* evidence of reduced artemisinin sensitivity in isolates obtained from patients with reduced *in vivo* response rates that could have suggested a gradual selection of ‘less-sensitive’ parasites. One explanation is that either unstable or *in vivo*-confined phenotypes may have escaped detection when measuring *in vitro* growth response rates in culture-adapted isolates. Alternatively, our *in vitro* test which covers >1 intraerythrocytic replication cycle may have been insensitive to changes in stage-specific artemisinin activity causing prolonged circulation of young ring-form parasites [33], [34]. Sustained parasite sensitivity to PPQ and LM (using assays previously shown to detect variation in sensitivity) [35]–[37] suggests that changes of treatment response rates were not due to parasite resistance to PPQ or LM and may help to explain the relatively small increase in parasite clearance times (46 hours in 2007–2008 up from 38 hours in 2005–2006). This conclusion is further supported by initial studies on piperaquine, which on its own can clear infections between 34 and 72 hours and by a recent meta-analysis showing accelerated clearance after treatment with ACTs containing novel drugs compared to compromised partner drugs such as chloroquine [38], [39]. Nevertheless, the confounding and interaction between immunity and potential change in parasite sensitivity in this study cautions against firm conclusions at this stage. We also cannot rule out the possibility that residual concentrations of former first-line drugs chloroquine or sulfadoxine-pyrimethamine contributed to faster parasite clearance during the first period of the study, that is, before the implementation of AM-LM as first-line treatment in 2006.

The observation of declining parasitological response to ACTs in the context of slowly declining malaria transmission intensity in Kilifi and minimal access to monotherapy raises a number of issues. First, immunity appears to play a complex role in parasite clearance during treatment. Second, it seems unlikely that parasites with reduced sensitivity to ACTs have spread from Southeast Asia to East Africa *en masse* to cause the apparently high prevalence of reduced sensitivity observed here. On the other hand, if tolerance is induced, rather than genetically encoded, for example through epigenetic mechanisms, its rise could be very rapid. Currently, we lack efficient molecular tools for mapping the spread of parasites with altered responsiveness and we do not know whether the observed small changes in responsiveness to treatment, which are reminiscent of long term observations from the Thai-Myanmar border [26] are precursors of worse-to-come artemisinin-specific “resistance” as reported from western Thailand [6]. At present, overall clinical response to ACTs remains adequate in Kenya, though decelerated parasite clearance *per se*, whether due to declining clinical immunity or changes in parasite sensitivity, could potentially impact on the clinical benefit of artemisinins in the treatment of malaria [40]. The observation of reducing response rates to ACTs on the Kenyan coast indicates an urgent need for surveillance of antimalarial drug efficacy in sentinel sites across Africa.

## Supporting Information

Figure S1 Dot plots of anti-*P. falciparum* schizont antibody responses (optical density) measured at baseline in children enrolled in 2005–2006 *vs.* 2007–2008. Solid lines represent medians and dashed lines indicate binomial 95% confidence intervals.
(EPS)

Figure S2 Fitted curves of the risk of residual parasitemia on day 1 in patients treated with DHA-PPQ (A–C) and AM-LM (D–F). The risk of day 1 parasitemia was fitted using logistic regression for baseline parasite density (A, D), patient age (B, E), and body-weight adjusted doses of DHA (C) and AM (F). Black lines represent predicted risk in patients enrolled in 2005–2006 and gray lines represent predicted risk in patients enrolled in 2007–2008.
(EPS)

Figure S3 Dot plots of half-maximal inhibitory concentrations (IC_50_) of culture-adapted *P. falciparum* isolates collected from patients in 2005–2006 and 2007–2008. IC_50_ responses were determined for dihydroartemisinin (DHA) (Fig. S3A), piperaquine (PPQ) (Fig. S3B) and lumefantrine (LM) (Fig. S3C).
(EPS)

Table S1 Relationship between pharmacokinetic and pharmacodynamic parameters over time.
(DOC)

Protocol S1 Trial Protocol.
(DOC)

Checklist S1 CONSORT Checklist.
(DOC)
