# Supplementary material for: Declining Responsiveness of Plasmodium falciparum Infections to Artemisinin-Based Combination Treatments on the Kenyan Coast
Source: PLoS One. 2011 Nov 10;6(11):e26005. doi: 10.1371/journal.pone.0026005 (PMC3213089; doi:10.1371/journal.pone.0026005)
Supplement: Table S1 — Relationship between pharmacokinetic and pharmacodynamic parameters over time. (DOC) [file pone.0026005.s004.doc]

**Table S1. Relationship between pharmacokinetic and pharmacodynamic parameters over time**

| **Pharmacokinetic-pharmacodynamic parameters** | **DHA-PPQ** | | | | | **AM-LM** | | | | |
| --- | --- | --- | --- | --- | --- | --- | --- | --- | --- | --- |
|  | **2005-2006** | | **2007-2008** | | **P value** | **2005-2006** | | **2007-2008** | | **P value** |
|  |  |  |  |  |  |  |  |  |  |  |
| **Day 7 serum concentration** | Piperaquine | | | | | Lumefantrine | | | | |
| N samples (%) ^1^ | 48/136 | (35%) | 57/81 | (70%) |  | 15/73 | (21%) | 86/160 | (54%) |  |
| Median conc. (range) in ng/mL | 43.3 | (11-149) | 43.3 | (14-166) | - | 536 | (178-3,270) | 391 | (56-2,560) | 0.2 |
| Proportion of patients with conc. <30ng/ML (PPQ) or <175mg/mL (LM) | 7/48 | (15%) | 12/57 | (21%) | 0.5 | 0/15 |  | 11/86 | (13%) | 0.4 |
| - with recrudescent infections | 0/47 | - | 2/57 | (4%) | - | 0/15 |  | 2/86 |  | - |
|  |  |  |  |  |  |  |  |  |  |  |
|  |  |  |  |  |  |  |  |  |  |  |
| **Dosing** | Dihydroartemisinin | | | | | Artemether | | | | |
| Median single dose (range) in mg/kg body weight | 2.2 | (1.5-3.2) | 2.0 | (1.5-3.2) | 0.05 | 2.0 | (1.4-3.1) | 1.8 | (1.3-3.1) | 0.004 |
| Day 1 parasite reduction ratio (range) |  |  |  |  |  |  |  |  |  |  |
| - in patients with DHA > 2.25 mg/kg  or AM > 2.0 mg/kg body weight ^2^ | 2.6 | (0.7-4.3) | 1.5 | (-0.1-4.0) | <0.001 | 2.2 | (0.6-3.7) | 1.7 | (-0.2-4.2) | 0.004 |
| - in patients with DHA ≤ 2.25 mg/kg  or AM ≤ 2.0 mg/kg body weight ^2^ | 2.3 | (0.6-4.2) | 1.8 | (0.6-4.1) | <0.001 | 1.7 | (0.7-3.9) | 1.4 | (-1.0-3.7) | <0.001 |

^1^ For piperaquine 5 samples had to be excluded because of incomplete regimen (4) or concentration below the LLOQ (1). For lumefantrine 4 samples were excluded because of concentration below the LLOQ (3) or incomplete regiment (1).

^2^ Target doses per single drug administration according to study protocol
