# Supplementary material for: Declining Responsiveness of Plasmodium falciparum Infections to Artemisinin-Based Combination Treatments on the Kenyan Coast
Source: PLoS One. 2011 Nov 10;6(11):e26005. doi: 10.1371/journal.pone.0026005 (PMC3213089; doi:10.1371/journal.pone.0026005)
Supplement: Protocol S1 — Trial Protocol. (DOC) [file pone.0026005.s005.doc]

**KENYA MEDICAL RESEARCH INSTITUTE**

**Centre for Geographic Medicine Research - Coast**

**Project proposal for consideration by the Scientific Steering Committee**

# TITLE OF PROJECT

A Phase III, randomized, non-inferiority trial, to assess the efficacy and safety of dihydroartemisinin-piperaquine (Artekin™) in comparison with artemether-lumefantrine (Coartem©) in children with uncomplicated *Plasmodium falciparum* malaria.

Protocol number: 1.0.6

# INVESTIGATORS AND INSTITUTIONAL AFFILIATIONS

## Investigators:

Dr. Steffen Borrmann^1,2^

Dr. Philip Sasi^1^

**Dr. Trudie Lang^1^**

Dr. Alexis Nzila^1^

**Dr. Simon Ndirangu^1^**

**Dr. Ahmed Abdallah^1^**

**Dr. Neema Mturi^1^**

**Mahfudh Bashraheil^1^**

Laura Mwalekwa^1^

Dr. Greg Fegan^1,3^

Dr. Michael Makanga^4^

Mr. Brett Lowe^1,2^

Dr. Pete Bull^1^

Dr. Norbert Peshu^1^

Prof. Kevin Marsh^1,2^

^1^ KEMRI, Centre for Geographic Medicine Research – Coast;

^2^ University of Heidelberg School of Medicine, Institute of Hygiene, Collaborative Research Network 544, Germany;

^3^ London School of Tropical Medicine and Hygiene, United Kingdom

^4^ European and Developing Countries Clinical Trials Partnership (EDCTP)

## Study Sponsor:

**University of Heidelberg School of Medicine, Heidelberg, Germany**

## Financial Support:

Dr. David Ubben (Programme Manager)

Medicine for Malaria Venture (MMV), Geneva, Switzerland

# TABLE OF CONTENT

1 TITLE OF PROJECT [1](#__RefHeading___Toc48636214)

2 INVESTIGATORS AND INSTITUTIONAL AFFILIATIONS [1](#__RefHeading___Toc48636215)

2.1 Investigators: [1](#__RefHeading___Toc48636216)

2.2 Study Sponsor: [1](#__RefHeading___Toc48636217)

2.3 Financial Support: [2](#__RefHeading___Toc48636218)

3 TABLE OF CONTENT [2](#__RefHeading___Toc48636219)

4 LIST OF ABBREVIATIONS [5](#__RefHeading___Toc48636220)

5 ABSTRACT [7](#__RefHeading___Toc48636221)

6 INTRODUCTION/BACKGROUND [7](#__RefHeading___Toc48636222)

7 JUSTIFICATION FOR THIS STUDY [9](#__RefHeading___Toc48636223)

8 NULL HYPOTHESIS [10](#__RefHeading___Toc48636224)

9 OBJECTIVES [10](#__RefHeading___Toc48636225)

9.1 Primary objectives: [10](#__RefHeading___Toc48636226)

9.2 Secondary objectives: [10](#__RefHeading___Toc48636227)

10 DESIGN AND METHODOLOGY [11](#__RefHeading___Toc48636228)

10.1 Study site [11](#__RefHeading___Toc48636229)

10.2 Study design [11](#__RefHeading___Toc48636230)

10.3 Study populations [11](#__RefHeading___Toc48636231)

10.3.1 Inclusion criteria [11](#__RefHeading___Toc48636232)

10.3.2 Exclusion criteria [12](#__RefHeading___Toc48636233)

10.4 Outcome measures and sample size. [12](#__RefHeading___Toc48636234)

10.4.1 End points [12](#__RefHeading___Toc48636235)

10.4.1.1 Primary end point [12](#__RefHeading___Toc48636236)

10.4.1.2 Secondary end points [13](#__RefHeading___Toc48636237)

10.4.2 Sample size calculation [13](#__RefHeading___Toc48636238)

10.5 Recruitment and trial procedures [14](#__RefHeading___Toc48636239)

10.5.1 Study visits [16](#__RefHeading___Toc48636240)

10.5.1.1 Day 0: Screening/Treatment phase [16](#__RefHeading___Toc48636241)

10.5.1.2 Day 1: Treatment phase [17](#__RefHeading___Toc48636242)

10.5.1.3 Day 2: Treatment phase [17](#__RefHeading___Toc48636243)

10.5.1.4 Day 3: Follow-up phase [18](#__RefHeading___Toc48636244)

10.5.1.5 Day 7: Follow-up phase [18](#__RefHeading___Toc48636245)

10.5.1.6 Days 14 and 21: Follow-up phase [18](#__RefHeading___Toc48636246)

10.5.1.7 Day 28: Follow-up phase [19](#__RefHeading___Toc48636247)

10.5.1.8 Days 35, 42, 49, 56, 63, and 84: Follow-up phase [19](#__RefHeading___Toc48636248)

10.5.2 Blood sampling [19](#__RefHeading___Toc48636249)

10.5.3 Parasite genotyping [19](#__RefHeading___Toc48636250)

10.5.4 Assessment of chemosensitivity of fresh parasite isolates [20](#__RefHeading___Toc48636251)

10.5.5 Determination of the plasma concentration of piperquine at asexual breakthrough infections during follow-up [20](#__RefHeading___Toc48636252)

10.5.6 Detection of sub-patent asexual parasitaemia on days 7 and 14 to predict subsequent parasitological failure [20](#__RefHeading___Toc48636253)

10.5.7 Investigation of the relationship between *var* gene transcription patterns and late asexual parasite recurrence [21](#__RefHeading___Toc48636254)

10.6 Patient follow-up [22](#__RefHeading___Toc48636255)

10.7 Safety assessment and reporting of symptoms and serious adverse events [22](#__RefHeading___Toc48636256)

10.7.1 Adverse Events [22](#__RefHeading___Toc48636257)

10.7.1.1 Definition of an Adverse Event [22](#__RefHeading___Toc48636258)

10.7.1.2 Severity, Relationship of Event to Study Drug, and outcome [23](#__RefHeading___Toc48636259)

10.7.1.3 Definition of a Serious Adverse Event [24](#__RefHeading___Toc48636260)

10.7.1.4 Reporting of serious adverse events [24](#__RefHeading___Toc48636261)

10.7.1.5 Reporting of adverse events [25](#__RefHeading___Toc48636262)

10.8 Subject completion and withdrawal [25](#__RefHeading___Toc48636263)

10.8.1 Subject Completion [25](#__RefHeading___Toc48636264)

10.8.2 Subject Withdrawal from Study [26](#__RefHeading___Toc48636265)

10.8.3 Subject Replacement [26](#__RefHeading___Toc48636266)

10.8.4 Subject Withdrawal from Investigational Product [26](#__RefHeading___Toc48636267)

11 INVESTIGATIONAL PRODUCTS & CONCOMITANT MEDICATIONS AND NON-DRUG THERAPIES [27](#__RefHeading___Toc48636268)

11.1 Investigational products [27](#__RefHeading___Toc48636269)

11.1.1 Study medication and dosages [27](#__RefHeading___Toc48636270)

11.1.1.1 Drug Dispensing [27](#__RefHeading___Toc48636271)

11.1.1.2 Dihydroartemisinin-piperaquine (Artekin™) [27](#__RefHeading___Toc48636272)

11.1.1.3 Artemether-lumefantrine (Coartem©) [28](#__RefHeading___Toc48636273)

11.1.2 Masking of treatment allocation [29](#__RefHeading___Toc48636274)

11.1.3 Randomisation [29](#__RefHeading___Toc48636275)

11.1.4 Stopping rule/discontinuation criteria [29](#__RefHeading___Toc48636276)

11.1.5 Accountability procedures for the investigational drugs [29](#__RefHeading___Toc48636277)

11.2 Concomitant medication and non-drug therapies [30](#__RefHeading___Toc48636278)

11.2.1 Permitted Medications [30](#__RefHeading___Toc48636279)

11.2.2 Prohibited Medications [30](#__RefHeading___Toc48636280)

11.3 Treatment failure [30](#__RefHeading___Toc48636281)

11.3.1 Early Treatment Failure (ETF) [30](#__RefHeading___Toc48636282)

11.3.2 Late treatment failure (LTF) [30](#__RefHeading___Toc48636283)

11.3.2.1 Late Clinical Failure (LCF) [30](#__RefHeading___Toc48636284)

11.3.2.2 Late Parasitological Failure (LPF) [30](#__RefHeading___Toc48636285)

11.3.3 Rescue Therapy [31](#__RefHeading___Toc48636286)

12 DATA MANAGEMENT [31](#__RefHeading___Toc48636287)

12.1 Data Recording [31](#__RefHeading___Toc48636288)

12.2 Analysis Populations [31](#__RefHeading___Toc48636289)

12.2.1 Modified Intention to Treat (ITT) Population [31](#__RefHeading___Toc48636290)

12.2.2 Per Protocol (PP) Population (or Evaluable Population) [31](#__RefHeading___Toc48636291)

12.2.3 Safety population [31](#__RefHeading___Toc48636292)

12.3 Statistical Analysis [32](#__RefHeading___Toc48636293)

12.3.1 Primary end point analysis [32](#__RefHeading___Toc48636294)

12.3.2 Secondary end point analyses [32](#__RefHeading___Toc48636295)

12.3.2.1 Secondary efficacy end point analyses [32](#__RefHeading___Toc48636296)

12.3.2.2 Safety and tolerability [32](#__RefHeading___Toc48636297)

13 TIME FRAME/DURATION OF THE PROJECT [33](#__RefHeading___Toc48636298)

14 BUDGET [33](#__RefHeading___Toc48636299)

15 ETHICAL CONSIDERATIONS [34](#__RefHeading___Toc48636300)

15.1 National Ethical Committee [34](#__RefHeading___Toc48636301)

15.2 Informed consent [34](#__RefHeading___Toc48636302)

15.3 Study Monitoring [34](#__RefHeading___Toc48636303)

15.4 Data Safety Monitoring Board (DSMB) [34](#__RefHeading___Toc48636304)

15.5 Quality Assurance (QA) [35](#__RefHeading___Toc48636305)

16 EXPECTED APPLICATION OF RESULTS [35](#__RefHeading___Toc48636306)

17 ROLE OF INVESTIGATORS [35](#__RefHeading___Toc48636307)

18 REFERENCES [36](#__RefHeading___Toc48636308)

19 APPENDIX I [38](#__RefHeading___Toc48636309)

# LIST OF ABBREVIATIONS

| ACPR | Adequate Clinical and Parasitological Response |
| --- | --- |
| ACT | Artemisinin-based combination chemotherapy |
| AE | Adverse Event |
| ALT | Alanine aminotransferase |
| BP | Blood Pressure |
| CI | Confidence Interval |
| CRF | Case Report Form |
| CRO | Clinical Research Organization |
| ECG | Electrocardiogram |
| EDTA | Ethylenediaminetetraacetic Acid |
| ETF | Early Treatment Failure |
| FCT | Fever Clearance Time |
| GCP | Good Clinical Practice |
| GLP | Good Laboratory Practice |
| GMP | Good Manufacturing Practice |
| h | Hour |
| Hb | Haemoglobin |
| ICH | International Conference on Harmonization |
| IEC | Independent Ethics Committee |
| IRB | Institutional Review Board |
| ITT | Intention to treat |
| IU | International Unit |
| LCF | Late Clinical Failure |
| LPF | Late Parasitological Failure |
| MMV | Medicines for Malaria Venture |
| mg | Milligram |
| NA | Not Applicable |
| PCR | Polymerase Chain Reaction |
| PCT | Parasite Clearance Time |
| PP | Per Protocol |
| RBC | Red Blood Cells |
| R&D | Research and development |
| SAE | Serious Adverse Event |
| SAS | Statistical Analysis System |
| SP | Sulfadoxine-pyrimethamine |
| TDR | Tropical Disease Research |
| TF | Treatment Failure |
| URTI | Upper Respiratory Tract Infection |
| WBC | White Blood Cells |
| WHO | World Health Organization |

# ABSTRACT

Artekin™ is an artemisinin-based fixed-dose antimalarial drug combination developed in the 1990s in China. The active components are dihydroartemisinin and piperaquine. Piperaquine is a bisquinoline compound originally synthesized in France with high *in vitro* activity in the nanomolar range against multidrug resistant *Plasmodium falciparum*. Piperaquine has been used as monotherapy in China for over two decades. It has proved to be generally very well tolerated and effective although resistance to the monotherapy has developed until the early 1990s prompting its reformulation in combination with other drugs, including dihydroartemisinin. Dihydroartemisinin has already been extensively evaluated and shown to be safe – with no major identifiable adverse effects. The combination has become widely used in South East Asia and is now registered in Cambodia, China and Vietnam.

Several factors make this combination a promising development: Artekin™ is a fixed-dose co-formulation used as a once daily 3-day regimen, and this may improve adherence and ease of drug administration in comparison for instance to the more complex regimens of artemether-lumefantrine (Coartem©). It is also inexpensive in comparison with other artemisinin-based combination chemotherapies, e.g. Coartem© or artesunate-mefloquine with an estimated cost per adult treatment of approximately 1 USD. Finally, preliminary clinical trials of this treatment in more than 5,000 patients carried out in South East Asia have shown it to be well tolerated and highly effective on par with Coartem© and artesunate-mefloquine in areas of high-level multi-drug resistance of *P. falciparum*.

This study is part of a multicentre phase III trial aimed at evaluating the safety and efficacy of the newly manufactured GMP batch of Artekin™ in 1,500 African children with uncomplicated *P. falciparum* malaria. In Kilifi, 500 patients aged 6-59 months with uncomplicated *P. falciparum* malaria will be randomly allocated to receive either dihydroartemisinin-piperaquine or artemether-lumefantrine. The objective of the study is to evaluate the efficacy and safety of dihydroartemisinin-piperaquine and to demonstrate that dihydroartemisinin-piperaquine (1) is at least as effective as artemether-lumefantrine and (2) cures at least 90% of treated patients.

# INTRODUCTION/BACKGROUND

It is generally acknowledged that artemisinin-based combination therapies (ACTs) currently provide the best available treatment option for uncomplicated *P. falciparum* malaria [1]. They give fast therapeutic responses, provide high cure ratios with a short duration of treatment, reduce gametocyte carriage, provide mutual protection against the emergence of resistance, and they are well tolerated. On the north-western border of Thailand, which harbours some of the most multi-drug resistant *P. falciparum* strains in the world, the widespread use of ACTs since 1994 has led to a sustained reduction in the incidence of *P.* *falciparum* malaria, and a hitherto unseen reversal of mefloquine *in vitro* resistance [2]. One of the main obstacles to more widespread adaptation of the use of ACTs in malaria endemic areas has been cost. The two currently available ACTs, artesunate-mefloquine and artemether-lumefantrine, cost USD 3 and USD 2.4, respectively per adult treatment course (expensive for majority of patients or national health services in most endemic countries). Projected costs for other ACTs, which are not yet available, are similar (artesunate-pyronaridine) or even higher (artesunate-atovaquone-proguanil). Furthermore mefloquine is associated with nausea, dizziness, and dysphoria, and has not been adopted in African countries because of high variability in absorption rates, and thus, efficacy concerns in children. Artemether-lumefantrine is very well tolerated but needs to be given twice daily, and lumefantrine has poor oral bioavailability in the if not concurrently taken with fatty food. Chlorproguanil-dapsone-artesunate (CDA) is a possible option that may be relatively inexpensive, but has not yet undergone clinical trials (currently still in phase II). There is also some concern that, by the time CDA is registered, continued widespread use of sulphadoxine-pyrimethamine would have already selected for highly antifolate resistant parasites. Artesunate-amodiaquine is also relatively inexpensive, but is not co-formulated yet, and already has reduced efficacy in Africa because of rapidly emerging parasite resistance to amodiaquine.

The combination of dihydroartemisinin and piperaquine was originally developed in the 1980s in China. Dihydroartemisinin-piperaquine has been extensively evaluated in clinical trials in Thailand, Vietnam, Cambodia, and China [3-6]. Efficacy has been high and tolerability uniformly excellent in all trials in these multi-drug resistant areas including Cambodia and Vietnam [4, 5]. These trials have used a constant dose of piperaquine, but initially the co-formulation also included trimethoprim (CV8). This formulation (CV8) has been used extensively in Vietnam, where it is part of national antimalarial treatment policy. More recently the dose of dihydroartemisinin in dihydroartemisinin-piperaquine has been increased by 25%. This product (Artekin™) has been registered in China, Cambodia and Vietnam. The fixed-dose combination dihydroartemisinin-piperaquine will cost approximately USD 1.2 or less for an adult treatment course given over 48 hours (similar to CDA or artesunate-amodiaquine).

Piperaquine is an orally active bisquinoline discovered by Rhone-Poulenc in the early 1960s and developed for clinical use in China in 1973 [7-9]. The phosphate salt is water-soluble. *In vitro* testing in several laboratories has shown that piperaquine is approximately equivalent to chloroquine against sensitive parasites and significantly more effective than chloroquine against chloroquine-resistant *P. falciparum* strains*.* Like other members of this class of antimalarials piperaquine chemically is thought to inhibit bio-crystallisation of haem in the food vacuole of the parasite. The estimated terminal elimination half-life is approximately 14 days in children with uncomplicated *P. falciparum* [10-12].

The gastrointestinal side effects of piperaquine are similar to those of chloroquine but milder at total doses of less than 2 g. In any other aspect the toxicity profiles are similar. Piperaquine replaced chloroquine as recommended treatment for *P.* *falciparum* malaria in China in 1978. Over the next 14 years 214 tons of piperaquine phosphate equivalent to 140,000,000 adult doses, were used for mass prophylaxis and treatment. Thus, in terms of drug treatment administered, piperaquine is amongst the most widely used antimalarial drugs in the world. Surveillance at the time found no adverse events other than rare cases of rash.

Dihydroartemisinin is the active metabolite of artesunate and artemether, and manufactured as an oral antimalarial drug in its own right China [13]. It has equivalent clinical efficacy to the more widely used artesunate. The mode of antimalarial action of this class of compounds is thought to involve intraparasitic Fe^++^ catalysed production of carbon centred free radicals.

Dihydroartemisinin is absorbed and eliminated rapidly (t_1/2_ circa 45 min in adult patients with uncomplicated *P. falciparum* malaria) [14]. To date no significant resistance to the artemisinin class of antimalarials has been reported and stable resistance cannot be induced in the laboratory. These drugs are safe in clinical use; there is no evidence that they produce neurotoxicity [15]. The only confirmed reports of adverse effects concern rare idiosynchratic type 1 hypersensitivity reactions [16]. There is overwhelming evidence of selected brainstem neurotoxicity from animal experiments, especially with oil-soluble artemisinin derivatives and a recent report of irreversible hearing loss after treatment with artemether-lumefantrine is of concern. However, detailed neurological examinations including pure-tone audiometry and auditory-evoked brain stem responses (ABR) failed to detect significant differences between mostly adult patients orally treated with either artesunate or artemether and age- and sex-matched controls [17]. A post-mortem comparative study of adult patients treated with either quinine or artemether for severe malaria was also negative with regard to any histopathological correlates of neuronal damages [18]. In experimental animals with early pregnancies fetal toxicity leading usually to fetal resorption occurs with high doses of the artemisinin derivatives. Clinical experience in pregnancy to date, however, has shown no cause for concern [19]. A recent WHO consensus committee concluded these drugs could be used in the second and third trimesters, but pending further information, not in the first trimester of pregnancy [20].

Dihydroartemisinin-piperaquine is co-formulated as a film tablet containing 40 mg of dihydroartemisinin and 320 mg of piperaquine phosphate for oral treatment of uncomplicated *P. falciparum* malaria. Dose finding studies in Thailand and Vietnam established the excellent safety and high efficacy of a once daily 3-day regimen of dihydroartemisinin at 4 mg/kg/day [21]. These phase II-type studies were conducted with the currently available non-GMP formulation in the expectation that oral bioavailability of the new GMP formulation will be similar. We will use the new GMP formulation provided by SigmaTau (Italy) in this study. Our study will be part of a phase III multicentre trial involving five studies sites in five African countries.

Piperaquine has a long terminal elimination half-life of approximately 14 days [11]. This might favour an accelerated emergence and spread of resistant parasite alleles, but by acting as a selective filter at time of reinfection [22].

Dihydroartemisinin-piperaquine is an ACT that seems to offer a highly efficacious, safe, well tolerated, simple and affordable treatment option for uncomplicated *P. falciparum* malaria in endemic countries.

# JUSTIFICATION FOR THIS STUDY

The inexorable spread of resistance of the malaria parasite *P. falciparum* to affordable antimalarial drugs such as chloroquine and sulfadoxine-pyrimethamine causes a public health crisis in sub-Saharan Africa. In the absence of a registered and effective malaria vaccine in the foreseeable future, antimalarial chemotherapy has to be improved dramatically to replace the currently used antimalarials. WHO currently advocates the use of artemisinin-based combination chemotherapy with prompt antiparasitic action and mutual protection against parasite resistance for the treatment of malaria [23, 24]. At present, the only fixed-dose combination registered up to international standards is artemether-lumefantrine (Coartem®), but artemether-lumefantrine has a rather complex treatment regimen requiring a total of six doses administered twice daily over three days, and the price is high (USD 2.4 under special agreements between the manufacturer and WHO). Other fixed-dose artemisinin-based combinations are under development (artesunate-amodiaquine, or chlorproguanil-dapsone-artesunate) but their future use might be compromised by existing cross-resistance to currently used antimalarial drugs (chloroquine and sulfadoxine-pyrimethamine). Dihydroartemisinin-piperaquine is a promising alternative, which has been shown very safe in more than 5,000 treated patients and proved to be highly effective in areas of multi-drug resistance. A fixed-dose co-formulation of dihydroartemisinin-piperaquine (Artekin™) is currently developed according to international standards as a once daily 3-day regimen, which is likely to improve adherence and to increase effectiveness. It is comparably inexpensive costing approximately 1 USD per adult treatment. If proved to be safe and efficacious, dihydroartemisinin-piperaquine will be an important addition to the currently available treatment options for uncomplicated childhood malaria due to multidrug-resistant *P. falciparum* in Kenya.

# NULL HYPOTHESIS

Dihydroartemisinin-piperaquine is non-inferior to artemether-lumefantrine in the treatment of paediatric patients aged 6 to 59 months with uncomplicated *P. falciparum* malaria as assessed by clinical and parasitological cure on day 28.

# OBJECTIVES

## Primary objectives:

The primary objective of the study is to measure the day 28, PCR-corrected cure ratios of dihydroartemisinin-piperaquine and artemether-lumefantrine and demonstrate that:

- The cure ratio of dihydroartemisinin-piperaquine is non-inferior to that of artemether-lumefantrine (non-inferiority margin = 5%);
- The cure ratio of dihydroartemisinin-piperaquine is at least 90%.

This cure ratio is defined as the proportion of evaluable patients with adequate clinical and parasitological response at day 28.

## Secondary objectives:

- PCR-uncorrected day 28 cure ratio;
- Safety profiles of the two treatments;
- Time to asexual parasite clearance (PCT);
- Time to fever clearance (FCT);
- Gametocyte prevalence and density on days 7, 14, 28, 42, 63 and 84;
- Haematological recovery (Haemoglobin (Hb) changes from day 0 to day 28, day 42, and day 84;
- Cure ratios at day 42 (PCR corrected and PCR uncorrected);
- Cure ratios at day 63 (PCR corrected and PCR uncorrected);
- Cure ratios at day 84 (PCR corrected and PCR uncorrected);
- Rate of PCR-confirmed reinfections to estimate the chemoprophylactic effect of dihydroartemisinin-piperaquine;
- Plasma concentration of piperaquine at time of asexual parasite reappearance;
- Detection of subpatent asexual parasitaemia on day 7 **and day 14;**
- **Assessment of the relationship between *var* gene transcription patterns and late asexual parasite recurrence**

# DESIGN AND METHODOLOGY

## Study site

The study will be conducted at the new Pingilikani study site, which is being extensively upgraded as an extension of the outpatient department (OPD) clinic at KEMRI Centre for Geographic Medicine Research-Coast (CGMR-C). The Pingilikani study site is located 20 km south of Kilifi town in Kilifi district along the Kenyan coastal region. It lies within the censused area of the Demographic Surveillance System operated by CGMR-C in Kilifi District. Good Clinical Practice will be implemented and adequately monitored. Severely-ill patents requiring hospitalisation can be transferred to Kilifi District Hospital at any times, day and night.

## Study design

This study is part of a phase III, randomized, open label, two-armed study involving 5 study centres, which will enroll a total 1,500 patients (1,000 dihydroartemisinin-piperaquine; 500 artemether-lumefantrine). Each study will enroll 300 patients into the multicentre study protocol (the other study sites are in Mozambique, Burkina Faso, Uganda, Zambia). In addition, 200 additional patients will be enrolled in the KEMRI study (1) to allow an independent statistical analysis of the KEMRI study and (2) to increase the precision of the estimated chemoprophylactic effect of dihydroartemisinin-piperaquine. Patients enrolled in the KEMRI study will also be followed up for a longer period until day 84 (instead of day 42). These changes will in effect act as an amendment to the multicentre study protocol and will not interfere with the analysis of the multicentre protocol. The data from the first 300 patients (day 0 - day 42) will be fully compatible with the multicentre protocol.

## Study populations

### Inclusion criteria

In order to be eligible, patients should satisfy the following inclusion criteria:

1. Males and Females aged between 6 months and 59 months inclusive.
2. Body weight of 5 Kg and above.
3. Microscopically confirmed, monoinfection of *Plasmodium falciparum* (parasitaemia ≥ 2,000/μL to 200,000/μL).
4. History of fever in the previous 24 hours or presence of fever (axillary temperature at ≥ 37.5°C).
5. Signed informed consent by the parents or guardians.
6. Parents’ or guardians’ willingness and ability to comply with the study protocol for the duration of the trial.

### Exclusion criteria

1. Participation in any investigational drug study during the previous 30 days.
2. Known hypersensitivity to the study drugs.
3. Severe malaria.
4. Danger signs: not able to drink or breast-feed, vomiting (>twice in 24 hours), recent history of convulsions (>1 in 24h), unconscious state, unable to sit or stand.
5. ECG abnormality that requires urgent management.
6. Presence of intercurrent illness or any condition which in the judgment of the investigator would place the subject at undue risk or interfere with the results of the study.
7. Severe malnutrition (defined as weight for height <70% of the median NCHS/WHO reference).

## Outcome measures and sample size.

### End points

#### Primary end point

The primary end point will be the PCR-corrected adequate clinical and parasitological response (PCR corrected ACPR) at day 28.

ACPR is defined as the absence of parasitaemia on day 28 irrespective of the axillary temperature without previously meeting any of the criteria of early treatment failure or late clinical or parasitological failure.

Patients with late asexual parasite reappearance will be considered ACPR if the PCR analysis shows a new infection rather than a recrudescence.

The total treatment failure is defined according to the WHO criteria [25] as the sum of early and late treatment failures (for definitions see chapter 7.3 Treatment failure).

#### Secondary end points

The secondary end points will be:

1. Crude (PCR uncorrected) adequate clinical and parasitological response ratio at day 28 (PCR-uncorrected ACPR)
2. Cure ratios at days 42, 63, and 84 (PCR-corrected and PCR-uncorrected ACPR)
3. Fever clearance time (FCT)

Fever clearance time will be defined as the time (in hours) from the start of a patient’s treatment to the first consecutive axillary temperature measurements below 37.5°C (body temperature <37.5ºC for at least 48 hours).

1. Asexual parasite clearance time (PCT)

Asexual parasite clearance time will be defined as the time (in days) from the start of a patient’s treatment to 2 consecutive negative blood slides (collected at different days).

1. Gametocyte carrier rates and geometric mean densities (excluding negatives) will be compared on days 7, 14, 28 and 42.
2. Changes of haemoglobin (Hb) concentration from day 0 to days 28, 42, 63, and 84.
3. Safety profiles of the two treatments.
4. Adverse events, vital signs, blood chemistry and haematology, electrocardiogram (ECG) will be monitored and changes in relevant laboratory parameters will be assessed.
5. Rate of PCR-confirmed reinfections
6. Plasma concentration of piperaquine at time of asexual parasite reappearance.
7. Detection of subpatent asexual parasitaemia on day 7 **and day 14**
8. **Assessment of the relationship between *var* gene transcription patterns and late asexual parasite recurrence**

### Sample size calculation

The study is designed as a non-inferiority trial.

The opinion of physicians and experts in malaria treatment has been sought for defining the non-inferiority margin, and this has been set at 5%. In addition, the experts have agreed that, in order for the new treatment to be considered efficacious, its PCR-corrected cure ratio at day 28 should not be lower than 90%. Therefore, in the present trial, dihydroartemisinin-piperaquine will be considered efficacious if both the following conditions are met (see section 2.1):

(i) The PCR-corrected cure ratio at day 28 of dihydroartemisinin-piperaquine is not inferior to the cure rate of artemether-lumefantrine by 5% or more, and independently of the reference drug performance,

(ii) The cure rate of dihydroartemisinin-piperaquine is at least 90%.

In summary, for the non-inferiority analysis, the clinical and statistical specifications for the sample size computations are as follows:

- Primary end point = PCR-corrected cure ratio at day 28.
- Primary analysis = based on 97.5% one-sided confidence intervals for the difference in day 28 cure ratios.
- Alpha = 0.025 (one-sided).
- Power = 80%.
- Cure ratio at day 28 of artemether-lumefantrine = 95% (results from multicentre phase III trial of Coartem in children; Dr. Michael Makanga personal communication).
- Non-inferiority margin for the difference (test-reference) = -0.05%.
- Analysis will be per-protocol.

To demonstrate non-inferiority of Artekin compared to Coartem the study will require 235 patients per treatment group. To allow for an approximate 5% loss to follow-up rate, 250 patients per treatment group (500 patients in total) will be enrolled in the Kilifi study.

## Recruitment and trial procedures

The parents or guardians of paediatric patients aged 6-59 months who present to the Pingilikani Health Centre with symptoms suggestive of malaria and a documented, uncomplicated *P. falciparum* infection with an asexual parasite density of 2,000–200,000 parasites/µL, will be informed by the health care facility staff about the ongoing malaria clinical trial. They will be requested to freely consent to let their child participate in the trial. Unwillingness or inability to participate will result in the prescription and administration of the local standard treatment regime for uncomplicated *P. falciparum* malaria (currently 4 mg/kg/day of amodiaquine for 3 days). A screening record will be maintained at the site to log all patients considered as potential subjects into the study, and a recruitment record to log all patients recruited into the study.

The parents or guardians who express interest in participation of their child in the study will receive detailed explanations about the trial from the study staff. Specifically they will be informed that the safety, tolerability and efficacy of two antimalarial drugs will be compared and that their child will be randomly assigned to receive treatment with one of the two drugs (by chance). Finally, they will be asked to sign the informed consent form (see Appendix IV). The study will be explained in the patient’s preferred language (English, Swahili, Giryama), and written informed consent shall be obtained on approved forms (accuracy of translation to be checked by back-translating into English).

At enrolment, children will be assigned a sequential study number. During the first 3 study days (days 0-2) patients will be either be admitted for observation and administration of study medication or managed as outpatients requiring home visits for supervised administration of study drugs at nighttime. Parents/guardians will then be encouraged to return to the clinic for follow up assessments on days 3, 7, 14, 21, 28, 35, 42, 63, and 84 and on any unscheduled day if the child is not well. Compensation for transport to and from the clinic will be provided. All medication will be administered under observation by an authorised member of the study team (physician, clinician, or study nurse).

Standard Operation Procedures (SOP) will be implemented according to international guidelines for the conduct of clinical research (Good Clinical Practice/International Conference on Harmonisation). A Quality Assurance (QA) programme will verify the quality of the trial procedures and data collection. In addition, the study will be independently monitored by a commercial Clinical Research Organisation (CRO; MDS Pharma, South Africa) on a fortnightly basis.

Patients will be assessed as summarized in the following flow-chart.

Table 1. Study Flow Chart of trial assessments and procedures.

|  | **Study days** | | | | | | | | | | | | | | |
| --- | --- | --- | --- | --- | --- | --- | --- | --- | --- | --- | --- | --- | --- | --- | --- |
|  | **0** | **1** | **2** | **3** | **7** | **14** | **21** | **28** | **35** | **42** | **49** | **56** | **63** | **84** | **Recurrent parasitaemia** |
| **Demographic data/medical history** | x |  |  |  |  |  |  |  |  |  |  |  |  |  |  |
| **Informed consent recording** | x |  |  |  |  |  |  |  |  |  |  |  |  |  |  |
| **Physical examination** | x | x | x | x | x | x | x | x | x | x | x | x | x | x | x |
| **Vital signs and weight^1^** | x^1^ | x | x | x | x | x | x | x | x | x | x | x | x | x | x |
| **Blood smear** | x | x | x | x | x | x | x | x | x | x | x | x | x | x | x |
| **Haematology** | x |  |  | x | x | x^3^ |  | x |  | x |  |  |  | x | x |
| **Clinical chemistry** | x |  |  | x | x^2^ | x^3^ |  | x |  | x |  |  |  |  | x |
| **Adverse events recording** | x | x | x | x | x | x | x | x | x | x | x | x | x | x | x |
| **Genotyping sampling** | x |  |  |  |  | x | x | x | x | x | x | x | x | x | x |
| **In vitro adaptation and drug assay** | x^4^ |  |  |  |  |  |  |  |  |  |  |  |  |  | x |
| **Piperaquine plasma concentration** |  |  |  |  |  |  |  |  |  |  |  |  |  |  | x^6^ |
| **Detection of subpatent asexual parasitaemia** |  |  |  |  | x^7^ | x^7^ |  |  |  |  |  |  |  |  |  |
| **Study of *var* gene expression** | **x** |  |  |  |  |  |  |  |  |  |  |  |  |  | **x** |
| **Concomitant treatments recording** | x | x | x | x | x | x | x | x | x | x | x | x | x | x | x |
| **Study medications** | x | x | x |  |  |  |  |  |  |  |  |  |  |  |  |

1. Weight will be measured at day 0

2. If abnormal at day 3

3. If abnormal at day 7

4. Using isolates from 50 randomly selected patients

5. If abnormal on day 28

6. Corresponding number of samples will be obtained from control (asexual parasite free) patients

7. Retrospective analysis only in case of recurrent recurrent asexual parasitaemia using samples taken for haematology

### Study visits

In the event of a suspected adverse drug reaction (irrespective of the day upon which this is noted) the physician shall seek permission to draw a venous blood sample, which will be used for a full blood count and biochemistry analysis.

#### Day 0: Screening/Treatment phase

1. Informed Consent

A signed informed consent from the parent/guardian shall be obtained before any tests or evaluations related to the study eligibility are carried out.

1. Demographic Data and Medical History

Demographic data and a general history of past and present illnesses will be recorded.

1. Physical and Clinical Examination

A general physical examination will be performed (see Appendix I).

A clinical examination will be performed (see Appendix I): symptoms, axillary or tympanic temperature (electronic thermometer). Weight and height will also be measured.

1. Vital Signs

Vital signs (systolic and diastolic blood pressure, heart rate).

1. PCR

A blood sample will be collected on filter paper (grade 1) for parasite genotyping.

1. Blood Slide

A thick and thin blood smear will be obtained from the subject to verify the presence of *P. falciparum* and to determine asexual and sexual parasite densities. Thick and thin blood films will be prepared, dried and stained with Giemsa stain according to standard operating procedures.

Parasite density will be calculated by counting the number of asexual parasites per 200 leukocytes in the thick blood film, based on an assumed leukocyte count of 8,000 /µl by light microscopy at 1000x magnification. One hundred high-powered fields (HPF) will examined (independent of presence or absence of asexual parasite stages). The parasite density per µL will be calculated using the following formula: numbers of parasites/µl = number of parasites counted x 8,000/number of leukocytes counted

1. Laboratory Tests

Blood haemoglobin, red blood cell count, total leukocyte count, differential count (neutrophils, basophils, eosinophils, monocytes, lymphocytes) and platelet count.

Total bilirubin, ALT and creatinine will be measured. **Parasite RNA will be analysed for variant gene expression patterns.**

1. Administration of the study drugs

For details see chapter 7 (Investigational product(s) & concomitant medications and non-drug therapies).

#### Day 1: Treatment phase

1. Physical and Clinical Examination

A general physical examination and a clinical examination will be performed: symptoms, axillary or tympanic temperature (electronic thermometer).

1. Vital Signs

Vital signs (systolic and diastolic blood pressure, heart rate) will be measured.

1. Blood Slide

A thick and thin blood smear will be obtained to verify the presence and to determine the density of asexual and sexual stages of *P*. *falciparum*.

1. Concomitant Pharmacological Treatments

Concomitant medications being taken by the patients will be recorded. For a list of allowed and disallowed medications, see section 4.

1. Adverse Events Report

The start and end of clinical adverse events will be recorded.

1. Administration of the study drugs

#### Day 2: Treatment phase

1. Physical and Clinical Examination

A general physical examination and a clinical examination will be performed: symptoms, axillary or tympanic temperature (electronic thermometer).

1. Vital Signs

Vital signs (systolic and diastolic blood pressure, heart rate) will be measured.

1. Blood Slide

A thick and thin blood smear will be obtained to determine the presence and the density of asexual and sexual stages of *P*. *falciparum*.

1. Concomitant Pharmacological Treatments

Concomitant medications being taken by the patients will be recorded. For a list of allowed and disallowed medications, see section 4.

1. Adverse Events Report

The start and end of clinical adverse events will be recorded.

1. Administration of the study drugs

#### Day 3: Follow-up phase

1. Physical and Clinical Examination

A general physical examination and a clinical examination will be performed: symptoms, axillary or tympanic temperature (electronic thermometer).

1. Vital Signs

Vital signs (Systolic and Diastolic Blood Pressure, Heart Rate) will be collected.

1. Blood Slide

A thick and thin blood smear will be obtained to determine the presence and the density of asexual and sexual stages of *P*. *falciparum.*

1. Concomitant Pharmacological Treatments

Concomitant medications being taken by the patients will be recorded. For a list of allowed and disallowed medications, see section 4.

1. Adverse Events Report

The start and end of clinical adverse events will be recorded.

1. Laboratory Tests

Blood haemoglobin, red blood cell count, total leukocyte count, differential count (neutrophils, basophils, eosinophils, monocytes, lymphocytes) and platelet count.

Total bilirubin, ALT and creatinine will be measured.

#### Day 7: Follow-up phase

During this visit, the same procedures as the visit 4 will be applied. EDTA and RNA samples will be taken for haematology and detection of subpatent parasitaemia, respectively. Laboratory tests will be performed in case of abnormality at D3.

#### Days 14 and 21: Follow-up phase

During these visits, the same procedures as the visit 4 will be applied.

A blood sample will be collected on filter paper (grade 1) for parasite genotyping **and a RNA sample for detection of subpatent parasitaemia.**

Laboratory tests will be performed at D14 in case of abnormality at D7.

#### Day 28: Follow-up phase

1. Physical and Clinical Examination

A general physical examination and a clinical examination will be performed: symptoms, axillary or aural temperature (electronic thermometer).

1. Vital Signs

Vital signs (Systolic and Diastolic Blood Pressure, Heart Rate) will be collected.

1. Blood Slide

A thick and thin blood smear will be obtained to determine the presence and the density of asexual and sexual stages of *P*. *falciparum.*

Filter paper for PCR analysis.

1. PCR

A blood sample will be collected on filter paper (grade 1) for parasite genotyping.

1. Laboratory Tests

Blood haemoglobin, red blood cell count, total leukocyte count, differential count and platelet count.

Total bilirubin, alanine aminotransferase (ALT) and creatinine will be measured.

The Investigator(s) will check all laboratory tests results and report them onto the Case Report Form (CRF) and will indicate those with potential clinical relevance. An abnormal laboratory test value of potential clinical relevance will be reported using the Adverse Event Form attached to the CRF.

1. Concomitant Treatments

Concomitant medications being taken by the patients will be recorded. For a list of allowed and disallowed medications, see section 4.

1. Adverse Events Report

The start and end of clinical adverse events will be recorded.

#### Days 35, 42, 49, 56, 63, and 84: Follow-up phase

During this visit, the same procedures as on days 14 and 21 will be performed.

However, haematology will be done on days 42 and 84 PCR on all days **and parasite RNA analysed for variant gene expression pattern**.

### Blood sampling

2 mL is required for each biochemistry assessment, 3 ml drug assays (piperaquine, lumefantrine, dihydroartemisinin) in a subset of patients, 1.2 mL for haematology, 1.6 ml for detection of subpatent asexual parasitaemia on days 7 **and 14**, 0.8 ml for parasite RNA at baseline, 2 ml for determination of piperaquine plasma level at asexual parasite reappearance, 50 µL for parasitology, and 50 µl for parasite genotyping. Thus, the total (maximum) blood volume collected per patient over a period of 84 days (approx. 3 months) should not exceed 26.8 ml and will average 18.0 ml (divided into 7 samples). Drug assays will be carried out as part of Dr. Alexis Nzila's project. Detailed information regarding this project will be submitted in parallel for clearance to the Ethics and Scientific Committees under the title: `Understanding the mechanisms of the antimalarial piperaquine resistance´.

### Parasite genotyping

Fifty µl of capillary blood obtained by finger stick will be spotted onto filter paper (labelled with study number of patient, study day and date) and dried before sealing it in a plastic bag. Each specimen will be recorded in a specimen logbook and stored at room temperature. The date of dispatch of all specimens will also be recorded. Pairs of parasite isolates from day 0 and day of recurrent asexual parasitaemia will be subjected to genotyping analysis to distinguish recrudescent from new infections. We will use a standard protocol based on the amplification and subsequent size differentiation via gel electrophoresis of three polymorphic *P. falciparum* genes: merozoite surface proteins-1 and –2 genes (*msp1* and *msp2*) and the glutamate-rich protein (*glurp*) gene. In addition, we will apply microsatellite analysis to confirm the results of the standard parasite genotyping approach described above [26].

### Assessment of chemosensitivity of fresh parasite isolates

The remaining pellet from the 2 ml sample taken for clinical chemistry (following centrifugation and removal of the plasma for the clinical chemistry tests) will be used for the *in vitro* tests. Subsequently, the leukocytes will be also be removed. After washing the cells for 3 times with 2 V of complete RPMI medium containing AB serum will be added to the red blood cells and centrifuge at 2000 rpm for 5 min. After removing the supernatant, this step will be repeated 2 more times. The resulting pellet will be re-suspended in a 1 V RPMI medium and a thin film will be made to check the parasitaemia. If the parasitaemia is >0.5 %, flesh erythrocytes will be added to bring down the parasitaemia to 0,5%. The haematocrit will be adjusted to 5% with RPMI. A volume of 200 μl of this suspension will be added into each well of the 96-well plate containing 50 μl of the drug solution (in RMPI), at ascending concentrations of drugs. The plate will be incubated for 48 hours at 37 ºC. Thereafter, radiolabelled hypoxanthine-(H^3^) will be added and the plates incubated for another 18 hours before assessing the parasite growth by radioactivity counting.

### Determination of the plasma concentration of piperquine at asexual breakthrough infections during follow-up

Breakthrough infections (recrudescent or new infections) from patients treated with dihydroartemisinin-piperaquine will be characterized in terms of piperaquine plasma levels at time of asexual parasite reappearance to analyse the relationship of drug selection pressure and the emergence of parasite resistance to piperaquine. The analysis will include (1) samples obtained from patients with subsequent asexual parasite reappearance due to recrudescence or reinfection and (2) a corresponding number of random samples obtained from patients without such an event during follow-up (total number of patients with breakthrough infections is estimated at approx. 50). These pharmacokinetic analyses will be performed in collaboration with Prof. Gilbert Kokwaro, KEMRI/Wellcome Trust Research Programme Nairobi and Dr. Niklas Lindegardh, Mahidol University Bangkok, Thailand.

### Detection of sub-patent asexual parasitaemia on days 7 and 14 to predict subsequent parasitological failure

Samples collected for day 7 haematology **and day 14** will be stored at -80ºC. Total RNA will be extracted (1) from samples obtained from patients with subsequent asexual parasite reappearance due to recrudescence or reinfection and (2) from a corresponding number of random samples obtained from patients without such an event during follow-up (total number of patients with breakthrough infections is estimated at approx. 50). The sensitivity of the subsequent analytical step will be increased by unspecifically amplifying extracted total RNA. Reverse transcriptase−polymerase chain reaction (RT-PCR) will then be used to detect transcripts of the ring-infected erythrocyte surface antigen, which exhibits asexual stage-specific expression. These investigations will be conducted in collaboration with the University of Heidelberg, Germany.

### Investigation of the relationship between *var* gene transcription patterns and late asexual parasite recurrence

**RNA will be collected in Roche RNA/DNA Stabilization Reagent for Blood/Bone Marrow. mRNA will be extracted using Roche mRNA Isolation Kit for Blood/Bone Marrow. The RNA analysis method follows previously described protocols (Bull et al., 2005; Kyes et al., 2000). Briefly, cDNA will be prepared from DNA-depleted total RNA DBLα sequences will be amplified using a primer annealing to the semi-conserved 5’ and 3’ ends of DBLα regions. Following PCR, purified DBLα sequences will be ligated into an appropriate vector and used to transform TOP10 cells. From each clinical isolate we aim to sequence approximately 50 cDNA DBLα clones. Sequencing will be carried out using an Applied Biosystems sequencing system. The expression patterns of *var* genes between pairs of isolates from baseline and asexual parasite recurrence will be examined for switches in dominant *var* gene transcripts. The equipment for the required high throughput sequencing is currently not yet available in Kenya and samples will therefore be analysed by the collaborating institution in the United Kingdom (Wellcome Trust Sanger Institute, Cambridge, UK). Samples will be stored at -80ºC until analysis. Storage of samples beyond the analysis stage is not planned.**

## Patient follow-up

Patients will be monitored daily until parasite and fever clearance is achieved. Active follow-up will be on days 3 and 7, then weekly on days 14, 21, 28, 35, 42, 56, and 63 and finally again on day 84; all subjects will be seen on any day between 0 and 84 upon request. The patient-specific CRF will be used to record clinical and laboratory data on each on these visits.

## Safety assessment and reporting of symptoms and serious adverse events

Safety and tolerability of the treatments will be evaluated by recording Adverse Events (AEs) and grading, laboratory, ECG and vital signs evaluations.

### Adverse Events

At each visit, the Investigator will ascertain the occurrence of any adverse events since the last visit. Any event must be recorded on the CRF.

#### Definition of an Adverse Event

An AE is any untoward medical occurrence in a patient or clinical investigation subject administered a pharmaceutical product and which does not necessarily have a causal relationship with this treatment.

An AE can therefore be any unfavourable and unintended sign (that could include a clinically significant abnormal laboratory finding), symptom or disease temporally associated with the use of a medicinal product, whether or not considered related to the medicinal product.

#### Severity, Relationship of Event to Study Drug, and outcome

The severity of an adverse event is to be scored according to the following scale:

1. Mild Awareness of sign or symptom, but easily tolerated
2. Moderate Discomfort enough to cause interference with usual activity
3. Severe Incapacitating with inability to work or perform usual activity

The relationship of an adverse event to study drug is to be assessed according to the following definitions:

1. Definitely unrelated Should be reserved for those events, which occur prior to test drug administration (e.g., washout or single-blind placebo) or for those events, which cannot be even remotely related to study participation (e.g. injury caused by a third party).
2. Unlikely There is no reasonable temporal association between the study drug and the suspected event and the event could have been produced by the subject's clinical state or other modes of therapy administered to the subject.
3. Possible The suspected adverse event may or may not follow a reasonable temporal sequence from study drug administration but seems to be the type of reaction that cannot be dismissed as unlikely. The event could have been produced or mimicked by the subject's clinical state or by other modes of therapy concomitantly administered to the subject.
4. Probable The suspected adverse event follows a reasonable temporal sequence from study drug administration, abates upon discontinuation of the drug, and cannot be reasonably explained by the known characteristics of the subject's clinical state.
5. Definitely related Should be reserved for those events, which have no uncertainty in their relationship to test drug administration: this means that a rechallenge was positive.

The outcome of each AE must be assessed according to the following classification:

1. Completely recovered The patient has fully recovered with no observable residual effects
2. Not yet completely recovered Improvement in the patient’s condition has occurred, but the patient still has some residual effects
3. Deterioration The patient’s overall condition has worsened
4. Permanent damage The AE has resulted in a permanent impairment
5. Death The patient died as a consequence of to the AE
6. Ongoing The AE has not resolved and remains the same as at onset
7. Unknown The outcome of the AE is not known because the patient did not return for follow-up (lost to follow-up)

#### Definition of a Serious Adverse Event

A serious adverse event (experience) (SAE) or reaction is any untoward medical occurrence that at any dose:

- Results in death;
- Is life-threatening;
- Requires hospitalization (other than for drug administration) or prolongation of existing hospitalization;
- Results in persistent or significant disability/incapacity; or
- Is a congenital anomaly/birth defect.

It also includes other important medical events (jeopardising the subject or requiring intervention to prevent one of the other outcomes listed in the definition above).

#### Reporting of serious adverse events

All serious adverse events, whether or not deemed drug-related, or expected, will be reported immediately or within 24 hours (one working day), using the Serious Adverse Event Notification Form, by facsimile or email to:

**Artekin group: Stefanie DeSantis**

**Sigma-Tau**

**Fax: +39-6-9139-3757**

**Email: Stefania.DeSantis@sigma-tau.it**

**Coartem group: Marc Cousin**

**Novartis**

**Email: marc.cousin@novartis.com**

Sigma-Tau and Novartis will be in charge of SAE notifications to relevant Regulatory Authorities.

In addition, all serious adverse events will be reported to:

Dr. Christine Wasunna

Secretary KEMRI/National Ethical Review Committee

Email: CWasunna@kemri.org

All other AEs not fulfilling the criteria of immediate reporting must be recorded on the Case Report Form.

#### Reporting of adverse events

For all adverse events identified, an adverse event report form will be completed.

For each possible adverse event identified and considered as serious, a serious adverse event notification form will be completed.

The following information will be recorded for all adverse events:

1. Description of event
2. Date of event onset
3. Date event reported
4. Severity of the event
5. Relationship of the event to study medication
6. Is the event serious?
7. Initials of the person reporting the event
8. Was the event episodic or intermittent in nature?
9. Outcome of adverse event
10. Action taken
11. Date event resolved.

A severity grading scale, based on toxicity grading scales developed by the WHO and the National Institutes of Health, Division of Microbiology and Infectious Diseases, will be used to grade severity of all symptoms, physical exam findings, and haemoglobin results (see Appendix I). Any new event, or an event present at baseline that is increasing in severity, will be considered as an adverse event.

## Subject completion and withdrawal

### Subject Completion

Subjects will be described as completing the study if they satisfied all study entry criteria, have received the treatment and have attended all visits prescribed by the protocol and provided blood samples as defined for these visits.

### Subject Withdrawal from Study

During the study, the following conditions are reasons for stopping the study treatment and/or for excluding the patients from further efficacy assessments:

1. Any treatment failure (including early failures);

2. Withdrawal of informed consent;

3. Use of antimalarial drugs (or antibiotics with antimalarial activity) outside of the study protocol;

4. Persistent vomiting of study drugs on day 0 (at least twice the same dose);

5. Failure to complete the study treatment;

6. Investigator’s request for safety reasons (for example, onset of serious adverse events, necessitating study interruption);

7. Lost-to-follow up: patient cannot be located by day 28.

Patients with one or more of the conditions listed above will have to be followed-up for the safety assessment until day 42.

These reasons for premature withdrawing will be referred to Day 28 (primary evaluation time). The reasons for withdrawing between Day 29 and Day 42 will also be recorded in the study CRF.

A protocol violation occurs when an event happens that does not allow for accurate interpretation of response to treatment.

Patients meeting one of the following criteria will be considered protocol violations but will not be withdrawn from follow-up:

- violation of any eligibility criteria;
- PCR unclassifiable results;
- failure to attend a follow-up visit on Days 0-2 and patient cannot be located within 6 hours of scheduled time;
- failure to attend two consecutive follow-up visits from Day 3 to Day 28 (visits from Day 3 to 28 must take place within 24 hours of the scheduled time).

The violations listed above are not valid reasons for withdrawing the patients from follow-up (both efficacy and safety assessments). On the contrary, the investigator must do his/her best to respect the allowed time windows at every visit, even if a violation occurred at a previous visit (i.e. a previous visit did not take place or occurred outside the allowed time window).

The protocol violations listed above will not be taken into account for establishing patient inclusion in the intention-to-treat population while they will be a reason for excluding the patients from the evaluable population.

### Subject Replacement

Subjects who are withdrawn during the study will be not be replaced.

### Subject Withdrawal from Investigational Product

Subjects who withdraw from taking the investigational product should be followed-up to monitor safety up until day 28 if possible, i.e. if not previously lost to follow-up.

# INVESTIGATIONAL PRODUCTS & CONCOMITANT MEDICATIONS AND NON-DRUG THERAPIES

## Investigational products

### Study medication and dosages

The study medications (Clinical Trial Material (CTM)) to be evaluated in this study will be prepared and provided by Sigma-Tau i.f.r. S.p.A., Pomezia (Rome), Italy.

The stability of the products used in the study will be determined in advance to assure suitability for use throughout the study. The results of these analyses will be included in the Trial Master File.

#### Drug Dispensing

The study medication will be administered at the clinic or at home by authorized members of the study team (mostly by a study nurse). Study medications given to infants and young children (<2 years old) will be crushed, mixed with 50 ml water, and administered as a slurry if they are unable to swallow. Study medications administered to older children will be given as tablets or fractions of tablets to be taken orally with a glass of water. The study nurse or the investigator will directly observe the intake of study medications. Patients will be observed for one hour to ensure that the medications are not vomited or spat out. Any patient who vomits or otherwise expels the medication within 30 minutes of administration will be retreated with a second dose. If vomiting occurs between 30 and 60 minutes after treatment, only half of the original dose will be given.

#### Dihydroartemisinin-piperaquine (Artekin™)

The drug will be given once daily for 3 days.

Dosing schedule

Medications will be administered at the following times:

Day 0: hour “X”

Day 1: hour “X” + 24 hours

Day 2: hour “X” + 48 hours

Dosing table

One tablet of Artekin™ contains either 20 mg of dihydroartemisinin and 160 mg of piperaquine or 40 mg of dihydroartemisinin and 320 mg of piperaquine. The dosing will be based on bodyweight (assuming a linear relationship between key pharmacokinetic parameters and body weight).

| **Weight in kg** | **mg of dihydroartemisinin to be given daily** | **Dose of dihydroartemisinin as mg/kg/d** |
| --- | --- | --- |
| 4 - 6 | 10 (1/2 tablet with 20 mg) | 1.67 - 2.5 |
| 7 - 12 | 20 (1 tablet with 20 mg) | 1.67 - 2.86 |
| 13 - 23 | 40 (1 tablet with 40 mg) | 1.74 - 3.08 |
| 24 - 35 | 80 (2 tablets with 40 mg) | 2.29 - 3.33 |
| 36 - 75 | 120 (3 tablets with 40 mg) | 1.6 - 3.64 |

| **Weight in kg** | **mg of piperaquine to be given daily** | **Dose of piperaquine as mg/kg/d** |
| --- | --- | --- |
| 4 - 6 | 80 (1/2 tablet with 160 mg) | 13.36 - 20 |
| 7 - 12 | 160 (1 tablet with 160 mg) | 13.36 – 22.88 |
| 13 - 23 | 320 (1 tablet with 320 mg) | 13.92 – 24.64 |
| 24 - 35 | 640 (2 tablets with 320 mg) | 18.32 – 26.4 |
| 36 - 75 | 960 (3 tablets with 320 mg) | 12.8 – 29.12 |

#### Artemether-lumefantrine (Coartem©)

Artemether-lumefantrine (Coartem®, Norvatis Pharma, Basel Switzerland) is co-formulated as fixed-ratio film tablets containing 20 mg artemether and 120 mg lumefantrine. Dosing will follow the recommendations of the manufacturer.

Dosing schedule

Medications will be administered at the following times:

Day 0: hour “X”

Day 0: hour “X” + 8 hours

Day 1: hour “X” + 24 hours

Day 1: hour “X” + 36 hours

Day 2: hour “X” + 48 hours

- 5 - < 15 kg: 1 tablet per dose
- 15 - < 25 kg: 2 tablets per dose
- 25 - < 35 kg: 3 tablets per dose

Tablets will be given, whenever possible with food/drink ~100 ml of reconstituted full cream milk or breast milk as appropriate. In case the infant/child is not able to ingest tablets, the tablet(s) will be dissolved before administration.

### Masking of treatment allocation

Because of the clear differences in form and colour of the two drug preparations, blinding of the treatment will not be possible at the point of administration. The use of the double-dummy technique with placebos for dihydroartemisinin-piperaquine and for artemether-lumefantrine would result into the administration of an excessive number of tablets per patient. However, all laboratory analyses including PCR will be performed by staff that will be unaware of the treatment allocation.

### Randomisation

Patients will be randomly assigned to one of the two treatment arms (dihydroartemisinin-piperaquine or artemether-lumefantrine). Blinding in the randomization process will be attained by the use of sealed envelopes. A Standard Operating Procedure (SOP) will control the use of the sealed envelopes.

The randomization list (blocks of 9) will be generated by MDS Pharma Services, using the plan procedure of SAS.

Treatment allocation and administration of medications will be performed by the study nurse or by the investigator at the clinic or during a home visit.

### Stopping rule/discontinuation criteria

No further patients will be enrolled into the study until the Principal Investigator (PI), clinical monitor and the sponsor have evaluated details of any serious adverse event.

The study will be stopped if the serious adverse event is classified as a serious adverse reaction to the study drug as assessed by the PI, monitor and sponsors in accordance with the definition of Good Clinical Practice/International Conference on Harmonisation guidelines.

### Accountability procedures for the investigational drugs

The PI will ensure that all clinical supplies are properly received, stored and documented. Only authorized dispensing staff will handle the study medication. These dispensing staff will account for all used and unused drug supplies. A Drug Accountability Log will be recorded in the investigator’s file (entries consisting of subject number, initials, date, time, number of tablets; dated and initialised by authorised members of the study team). The remaining medication will be kept in the original blister packages and stored for clinical monitoring and drug accounting purposes.

All investigational products will be used only in accordance with the protocol: any discrepancy between drugs received, used and returned will need to be explained.

## Concomitant medication and non-drug therapies

### Permitted Medications

All concomitant medications taken during the study will be recorded in the CRF with indication, dose information, and dates of administration. All subjects may be given paracetamol as an analgesic during the study, at the discretion of the treating physician. Antibiotics without known antimalarial activity, e.g. penicillin, cephalosporins and aminoglycosides, can be prescribed.

### Prohibited Medications

Any antimalarial, or antibiotic with antimalarial activity (erythromycin and other macrolides, co-trimoxazole and other sulphonamides, any tetracycline (including doxycycline, and quinolones). Intake of such drugs will lead to the withdrawal of the patient from the study and classification as treatment failure in the intention-to-treat population.

## Treatment failure

A subject will be considered a treatment failure if they experience either early or late treatment failure based on the following definitions.

### Early Treatment Failure (ETF)

- Development of danger signs or severe malaria on days 0-3, in the presence of asexual parasitaemia,
- Parasite density on day 2 > day 0 count irrespective of axillary temperature,
- Presence of asexual parasitaemia on day 3 with fever (axillary temperature ≥ 37.5°C),
- Asexual parasitaemia on day 3 ≥ 25 % of count on day 0.

### Late treatment failure (LTF)

#### Late Clinical Failure (LCF)

- Development of danger signs or severe malaria after day 3 in the presence of parasitaemia,
- Presence of parasitaemia and fever on any day from day 4 to day 28, without previously meeting the criteria of ETF.

#### Late Parasitological Failure (LPF)

- Reappearance of asexual parasitaemia after initial clearance between day 4 and day 84 (identified as recrudescent infection by PCR analysis) in the absence of fever (axillary temperature <37.5°C) without previously meeting the criteria of ETF or LCF.

### Rescue Therapy

Atovaquone-proguanil (Malarone©; 20 and 8 mg/kg of body weight, respectively, administered once daily for 3 days) will be used as rescue treatment for both early and late treatment failures in both treatment arms with uncomplicated disease. Intravenous quinine and appropriate supportive care will be used for any cases that progress to severe malaria (requiring admission to the paediatric ward at Kilifi District Hospital).

# DATA MANAGEMENT

## Data Recording

A case-record form (CRF) will be completed for each patient documenting demographic and anthropometric data, symptoms prior to clinic attendance, concomitant illness, drug history, results of physical examinations, vital signs, laboratory results incl. parasitology, haematology and clinical chemistry, and study drug administration. All entries will only be made by authorized study team members and dated and initialized. CRFs will be kept in a secure area to ensure confidentiality of medical and personal data.

## Analysis Populations

### Modified Intention to Treat (ITT) Population

Defined as the ITT population with the exclusion of the lost-to-follow-up patients before Day 28 for unknown reasons (non informative drop-outs). More specifically, patients withdrawing from the study for reasons 7 will be excluded, while all other withdrawals will be counted as failures (these reasons might be considered potentially correlated with lack of efficacy, i.e. are referred to as informative drop-outs).

### Per Protocol (PP) Population (or Evaluable Population)

Defined as all randomized patients who will be eligible according to the study protocol, received at least 80% of the study medication, will not take other anti-malarial drugs and, in case of the presence of asexual parasite stages on thick or thin blood smears, will have an evaluable PCR analysis.

### Safety population

All subjects who have received any dose of the study medication will be included in the safety population. The analysis of the 28 and 63-day cure ratios will be performed both for the intention-to-treat (ITT) patient population (all randomised patients) and the evaluable patient population. Patients are evaluable for the analysis of the 28 and 63-day cure ratios if parasite counts are recorded up to Day 28 and 63 or the patient discontinues due to reappearance of *P. falciparum* confirmed by PCR genotyping to be recrudescent.

The ITT analysis also includes patients who discontinue before Days 28 and 63 due to other reasons. These patients will be counted in the ITT analysis as treatment failures regardless of their reason for discontinuation.

Safety and tolerability measurements will be summarised for the ITT patient population.

## Statistical Analysis

### Primary end point analysis

The primary analysis will be based on a 97.5% (one-sided) confidence interval (CI) computed on the difference between the 28-day PCR corrected cure ratios (PP population) of the test and the reference treatments, respectively.

In order to claim that dihydroartemisinin-piperaquine is efficacious, the lower limit of the 97.5% confidence interval of the difference between the treatment groups must be >-0.05 and the point estimate for the 28-day PCR corrected cure ratio of dihydroartemisinin-piperaquine must be >0.90%, in the PP population.

All patients withdrawn from the study will be considered as failures in the ITT population (worst-case scenario).

For computing the PCR-corrected cure ratio, the following rules will be applied:

- The patients with reappearing asexual parasitaemia, which are subsequently found to be due to re-infections are considered as treatment success in both PP and ITT populations;
- The patients for whom the PCR analysis is not interpretable (or blood sample unavailable) are considered as treatment failure in the ITT population, but are excluded from the PP population.

Robustness of results for the primary end point will be investigated by using other analyses, such as the survival analysis techniques.

### Secondary end point analyses

#### Secondary efficacy end point analyses

Normally distributed variables will be summarized by calculating means and standard deviations (SD) and compared with parametric tests (unpaired and paired Student’s *t* or analysis-of-variance (ANOVA) tests as appropriate). Non-normally distributed variables will be summarized by median and interquartile ranges (IQR) or geometric means and 95% confidence intervals. Comparisons will be made with nonparametric tests (unpaired and paired Wilcoxon or Kruskal-Wallis rank tests as appropriate). The Fisher’s exact test will be used to assess differences in categorical outcome variables between the cohorts. The statistical significance level will be set to 5%.

#### Safety and tolerability

The ITT population will be considered for the following analysis.

Adverse Events (AEs) will be tabulated according to degree of seriousness, time of treatment at AE onset, severity, relation to study drug, action taken, if any, and final outcome.

Vitals signs and ECG will be analysed descriptively.

ECG results will be classified as normal and abnormal. The evaluation of ECG and QT interval will be done according to the European Medicines Evaluation Agency (EMEA) directive “Point to consider: the assessment of the potential for QT interval prolongation by non-cardiovascular medicinal products”. EMEA (CPMP), London 17/12/1997 (CPMP/986/96).

# TIME FRAME/DURATION OF THE PROJECT

We plan to carry out the study from JULY 2005 to December 2007 (time from start from enrollment to last follow-up visit). Data analysis and report writing will commence in January 2007. We intend to submit the results of this study by April 2008 as an independent report to an international peer-reviewed scientific journal.

# BUDGET

*COE = cost of employment (includes benefits, e.g. gratuities, insurance and medical cover); TCOE = total cost of employment (includes annual salary and benefits)

The cost per patient is calculated by dividing the sum of the subtotals from the following budget lines: staff costs, clinical costs, transport costs, admission costs/meals and stationery/freight/communications (USD 201,998.30) by the total number of patients (n=500).

# ETHICAL CONSIDERATIONS

This study will be conducted in accordance with Good Clinical Practice/International Conference on Harmonisation (GCP/ICH) guidelines and all applicable regulatory requirements, including, the latest revision of the Declaration of Helsinki from the year 2000. A complete and fully referenced set of Standard Operating Procedures (SOP) directs the conduct of the clinical study. This set of SOP is linked to the SOPs for the KEMRI clinical laboratories at Kilifi District Hospital representing a comprehensive network of quality assurance measures.

## National Ethical Committee

The present protocol will be submitted to the Kenyan national ethical committee for approval. Copies of the written approval will be remitted to all partners involved in the study. During the study, the investigators are committed to inform the ethical committee of any emergent problems, serious adverse events, or protocol amendments.

## Informed consent

Informed consent will be obtained before the subject can participate in the study. Female patients of child bearing potential will first be required to give a written consent for a pregnancy test before consideration for possible participation in the study. The contents and process of obtaining informed consent will be in accordance with all applicable regulatory requirements. The consent form generated by the investigator with the assistance of EDCTP/WHO, should be approved (along with the protocol) by the ERC/IRB and be acceptable to EDCTP/WHO. Consent forms must be in a language fully comprehensible to the prospective patient, their relatives, guardians or, if necessary, legal representatives. Informed consent shall be documented by the use of a written consent form approved by the ERC/IRB and signed by the patient or the patient's legally authorised representative.

## Study Monitoring

An independent monitor (MDS Pharma, Johannesburg, South Africa) will verify the best conduct of the study through frequent contacts by phone and in person with the Principal Investigator and site staff, in accordance with the Standard Operating Procedures and Good Clinical Practice, with the purposes of facilitating the work and attaining the objectives of the study.

## Data Safety Monitoring Board (DSMB)

An independent Data Safety Monitoring Board will review relevant efficacy data and all safety data.

## Quality Assurance (QA)

A random sample of the blood smear readings and PCR analysis will be checked for accuracy by an independent laboratory.

# EXPECTED APPLICATION OF RESULTS

This is a pivotal phase III study on the combination of dihydroartemisinin-piperaquine, aimed at evaluating the efficacy and safety of the newly manufactured GMP batch of the co-formulated tablet in the treatment of African children with uncomplicated *P. falciparum* malaria. If dihydroartemisinin-piperaquine proves to be efficacious and safe, it will provide a valuable addition to the therapeutic armamentarium for the treatment of malaria in Kenya. Of particular interest is that this antimalarial has added advantages of being a fixed-dose artemisinin-based combination chemotherapy that is relatively inexpensive and has a simple dosage regimen. The results of this study will provide data for the registration of this promising antimalarial combination as a prerequisite for its potential use as a first-line antimalarial drug.

# ROLE OF INVESTIGATORS

Dr. Steffen Borrmann: principal investigator; design, conduct, analysis and reporting of study

Dr. Philip Sasi: co-ordinating, conduct, analysis and reporting of study

Dr. Trudie Lang: co-ordinating, conduct, analysis and reporting of study

Dr. Simon Ndirangu: responsable for pharmacokinetic analyses

Dr. Ahmed Abdallah: conduct, analysis and reporting of study

Dr. Neema Mturi: conduct, analysis and reporting of study

Mahfudh Bashraheil: conduct of study

Laura Mwalekwa: implementation of study

Dr. Greg Fegan: design, analysis and reporting of study

Dr. Michael Makanga: design of study

Mr. Brett Lowe: design, conduct, analysis and reporting of study

Dr. Pete Bull: supervision of variant gene expression studies, writing of scientific report

Dr. Alexis Nzila: design, conduct, analysis and reporting of study

Dr. Norbert Peshu: design, analysis and reporting of study

Prof. Kevin Marsh: design, analysis and reporting of study

# REFERENCES

1. WHO. WHO Technical Consultation. Antimalarial drug combination therapy: WHO/CDS/RBM/2001.35 April 2001, 2001

2. Nosten F, van Vugt M, Price R, et al. Effects of artesunate-mefloquine combination on incidence of Plasmodium falciparum malaria and mefloquine resistance in western Thailand: a prospective study. Lancet 2000;356:297-302

3. Guo XB. [Randomised comparison on the treatment of falciparum malaria with dihydroartemisinin and piperaquine]. Zhonghua Yi Xue Za Zhi 1993;73:602-4, 638

4. Denis MB, Davis TM, Hewitt S, et al. Efficacy and safety of dihydroartemisinin-piperaquine (Artekin) in Cambodian children and adults with uncomplicated falciparum malaria. Clin Infect Dis 2002;35:1469-76

5. Tran TH, Dolecek C, Pham PM, et al. Dihydroartemisinin-piperaquine against multidrug-resistant Plasmodium falciparum malaria in Vietnam: randomised clinical trial. Lancet 2004;363:18-22

6. Karunajeewa H, Lim C, Hung TY, et al. Safety evaluation of fixed combination piperaquine plus dihydroartemisinin (Artekin) in Cambodian children and adults with malaria. Br J Clin Pharmacol 2004;57:93-9

7. Qu FY, Huang WJ and Wang LQ. [Experimental studies on orally administered long-acting antimalarials. II. Observations on therapeutic and prophylactic effect of hydroxypiperaquine on rodent malaria (author's transl)]. Yao Xue Xue Bao 1981;16:298-301

8. Yang HL, Yang PF, Liu DQ, et al. [Sensitivity in vitro of Plasmodium falciparum to chloroquine, pyronaridine, artesunate and piperaquine in south Yunnan]. Zhongguo Ji Sheng Chong Xue Yu Ji Sheng Chong Bing Za Zhi 1992;10:198-200

9. Chen L, Qu FY and Zhou YC. Field observations on the antimalarial piperaquine. Chin Med J (Engl) 1982;95:281-6

10. Hung TY, Davis TM and Ilett KF. Measurement of piperaquine in plasma by liquid chromatography with ultraviolet absorbance detection. J Chromatogr B Analyt Technol Biomed Life Sci 2003;791:93-101

11. Hung TY, Davis TM, Ilett KF, et al. Population pharmacokinetics of piperaquine in adults and children with uncomplicated falciparum or vivax malaria. Br J Clin Pharmacol 2004;57:253-62

12. Davis TM, Hung TY, Sim IK, Karunajeewa HA and Ilett KF. Piperaquine: a resurgent antimalarial drug. Drugs 2005;65:75-87

13. Li GQ, Guo XB, Fu LC, Jian HX and Wang XH. Clinical trials of artemisinin and its derivatives in the treatment of malaria in China. Trans R Soc Trop Med Hyg 1994;88 Suppl 1:S5-6

14. Na-Bangchang K, Krudsood S, Silachamroon U, et al. The pharmacokinetics of oral dihydroartemisinin and artesunate in healthy Thai volunteers. Southeast Asian J Trop Med Public Health 2004;35:575-82

15. Taylor WR, White NJ. Antimalarial drug toxicity: a review. Drug Saf 2004;27:25-61

16. Leonardi E, Gilvary G, White NJ and Nosten F. Severe allergic reactions to oral artesunate: a report of two cases. Trans R Soc Trop Med Hyg 2001;95:182-3

17. Van Vugt M, Angus BJ, Price RN, et al. A case-control auditory evaluation of patients treated with artemisinin derivatives for multidrug-resistant Plasmodium falciparum malaria. Am J Trop Med Hyg 2000;62:65-9

18. Hien TT, Turner GD, Mai NT, et al. Neuropathological assessment of artemether-treated severe malaria. Lancet 2003;362:295-6

19. McGready R, Cho T, Keo NK, et al. Artemisinin antimalarials in pregnancy: a prospective treatment study of 539 episodes of multidrug-resistant Plasmodium falciparum. Clin Infect Dis 2001;33:2009-16

20. WHO. Assessment of safety of Artemisinin Compounds in Pregnancy. WHO TDR/RBM 2002, 2002

21. Ashley EA, Krudsood S, Phaiphun L, et al. Randomized, controlled dose-optimization studies of dihydroartemisinin-piperaquine for the treatment of uncomplicated multidrug-resistant falciparum malaria in Thailand. J Infect Dis 2004;190:1773-82

22. White NJ. Antimalarial drug resistance. J Clin Invest 2004;113:1084-92

23. Guerin PJ, Olliaro P, Nosten F, et al. Malaria: current status of control, diagnosis, treatment, and a proposed agenda for research and development. Lancet Infect Dis 2002;2:564-73

24. Olliaro P, Taylor WR and Rigal J. Controlling malaria: challenges and solutions. Trop Med Int Health 2001;6:922-7

25. WHO. Assessment and monitoring of antimalarial drug efficacy for the treatment of uncomplicated falciparum malaria. 2003

26. Su XZ, Carucci DJ and Wellems TE. Plasmodium falciparum: parasite typing by using a multicopy microsatellite marker, PfRRM. Exp Parasitol 1998;89:262-5

# APPENDIX I

**Criteria for Severe Malaria/Danger Signs**

**Severe Malaria**

- Unarousable coma *(if after convulsion, > 30 min)*
- Repeated convulsions *(> 2 within 24 h)*
  - Severe anaemia *(Hb < 5.0 g/dL)*
  - Respiratory distress *(laboured breathing at rest)*

**Danger Signs**

- Recent convulsions *(1-2 within 24 h)*
- Altered consciousness  *(confusion, delerium, psychosis)*
- Lethargy
  - Unable to drink or breast feed
  - Vomiting everything
  - Unable to stand/sit due to weakness
